# Early combination therapy of empagliflozin and linagliptin exerts beneficial effects on pancreatic β cells in diabetic *db/db* mice

**DOI:** 10.1038/s41598-021-94896-w

**Published:** 2021-08-09

**Authors:** Yoshiro Fushimi, Atsushi Obata, Junpei Sanada, Yuka Nogami, Tomoko Ikeda, Yuki Yamasaki, Yoshiyuki Obata, Masashi Shimoda, Shuhei Nakanishi, Tomoatsu Mune, Kohei Kaku, Hideaki Kaneto

**Affiliations:** 1 Department of Diabetes, Endocrinology and Metabolism, Kawasaki Medical School, 577 Matsushima, Kurashiki, 701-0192 Japan; 2 Department of Clinical Nutrition, Kawasaki University of Medical Welfare, 288 Matsushima, Kurashiki, 701-0193 Japan

**Keywords:** Molecular biology, Endocrinology

## Abstract

Effects of combination therapy of dipeptidyl peptidase-4 (DPP-4) inhibitor and sodium-glucose co-transporter 2 (SGLT2) inhibitor on β-cells are still unclear, although combination agent of these two drugs has become common in clinical practice. Therefore, we aimed to elucidate the effects of DPP-4 inhibitor and/or SGLT2 inhibitor on β-cell mass and function and compared their effects between in an early and advanced phase of diabetes. We used 7-week-old *db/db* mice as an early phase and 16-week-old mice as an advanced phase and treated them for 2 weeks with oral administration of linagliptin, empagliflozin, linagliptin + empagliflozin (L + E group), and 0.5% carboxymethylcellulose (Cont group). Blood glucose levels in Empa and L + E group were significantly lower than Cont group after treatment. In addition, β-cell mass in L + E group was significantly larger than Cont group only in an early phase, accompanied by increased Ki67-positive β-cell ratio. In isolated islets, mRNA expression levels of insulin and its transcription factors were all significantly higher only in L + E group in an early phase. Furthermore, mRNA expression levels related to β-cell differentiation and proliferation were significantly increased only in L + E group in an early phase. In conclusion, combination of DPP-4 inhibitor and SGLT2 inhibitor exerts more beneficial effects on β-cell mass and function, especially in an early phase of diabetes rather than an advanced phase.

## Introduction

Diabetes is the most prevalent metabolic disease, which causes serious complications such as micro and/or macroangiopathy. In addition, the number of patients with diabetes is still drastically increasing throughout the world. Type 2 diabetes mellitus (T2DM) is caused by insulin resistance in tissues such as the liver, skeletal muscle and adipose tissue accompanied with impaired pancreatic β-cell function^1–4^. It has been reported that β-cell mass in patients with T2DM is decreased compared to healthy subjects in some human studies^5,6^. Furthermore, it is also reported that in obese patients with impaired fasting glucose, β-cell mass is also decreased compared to normoglycemic subjects^5^. The United Kingdom Prospective Diabetic Study (UKPDS) has reported that deterioration of pancreatic β-cell function is already observed several years before the diagnosis of T2DM, which finally leads to progression of T2DM^7^. Therefore, how to protect pancreatic β-cell mass and/or function is a very important issue. Our group has reported that DPP-4 inhibitor (vildagliptin) protects pancreatic β-cell mass and function in KKAy mice and SGLT2 inhibitor (luseogliflozin) also protects β-cell mass and function in diabetic *db/db* mice. Especially, our group elucidated that luseogliflozin exerts more beneficial effects on β-cells when administered in an early phase of diabetes rather than in an advanced phase of diabetes^8–10^.

In addition to these backgrounds, DPP-4 inhibitors have become commonly used in many patients with T2DM recently as they exert good glucose-lowering effect with low risk of hypoglycaemia. DPP-4 inhibitors prevent cleavage and degradation of GLP-1 and promote glucose- or nutrient-stimulated insulin secretion and suppression of glucagon secretion^11^. On the other hand, SGLT2 inhibitors reduce renal glucose reabsorption from the renal proximal tubule and increase urinary glucose excretion, which is a totally different mechanism compared with DPP-4 inhibitors^12^. SGLT2 inhibitors are very promising as they improve glucose toxicity, reduce body weight and ameliorate insulin resistance^13–15^. In addition, large cardiovascular outcome trials have shown that SGLT2 inhibitors improve 3-point major adverse cardiovascular events (MACE) and renal outcomes^16–18^. It is notable that such effects were not observed in DPP-4 inhibitors. It seems that the combination therapy of DPP-4 inhibitors and SGLT2 inhibitors is very promising as both inhibitors have totally different mechanisms for amelioration of hyperglycemia with the pretty low risk of hypoglycaemia. In fact, the efficacy and safety of combination therapy in Japanese subjects with T2DM have been shown in large clinical trials^19–22^.

In addition, combination drugs of both inhibitors became commercially available in clinical practice very recently and it is drawing much attention from the point of its cost-effectiveness and improvement of patients’ adherence. However, it is still poorly understood whether combination therapy of DPP-4 inhibitors and SGLT2 inhibitors has more beneficial effects on β-cell mass and function compared to each medication and whether there are any different effects on pancreatic β-cells when these medications are administered between in an early phase and an advanced phase of diabetes. Therefore, we aimed to address these questions in this study.

## Results

### Empagliflozin and combination therapy significantly reduced blood glucose levels and linagliptin and combination therapy significantly increased active GLP-1 levels in diabetic *db/db* mice

**Figure 1:**
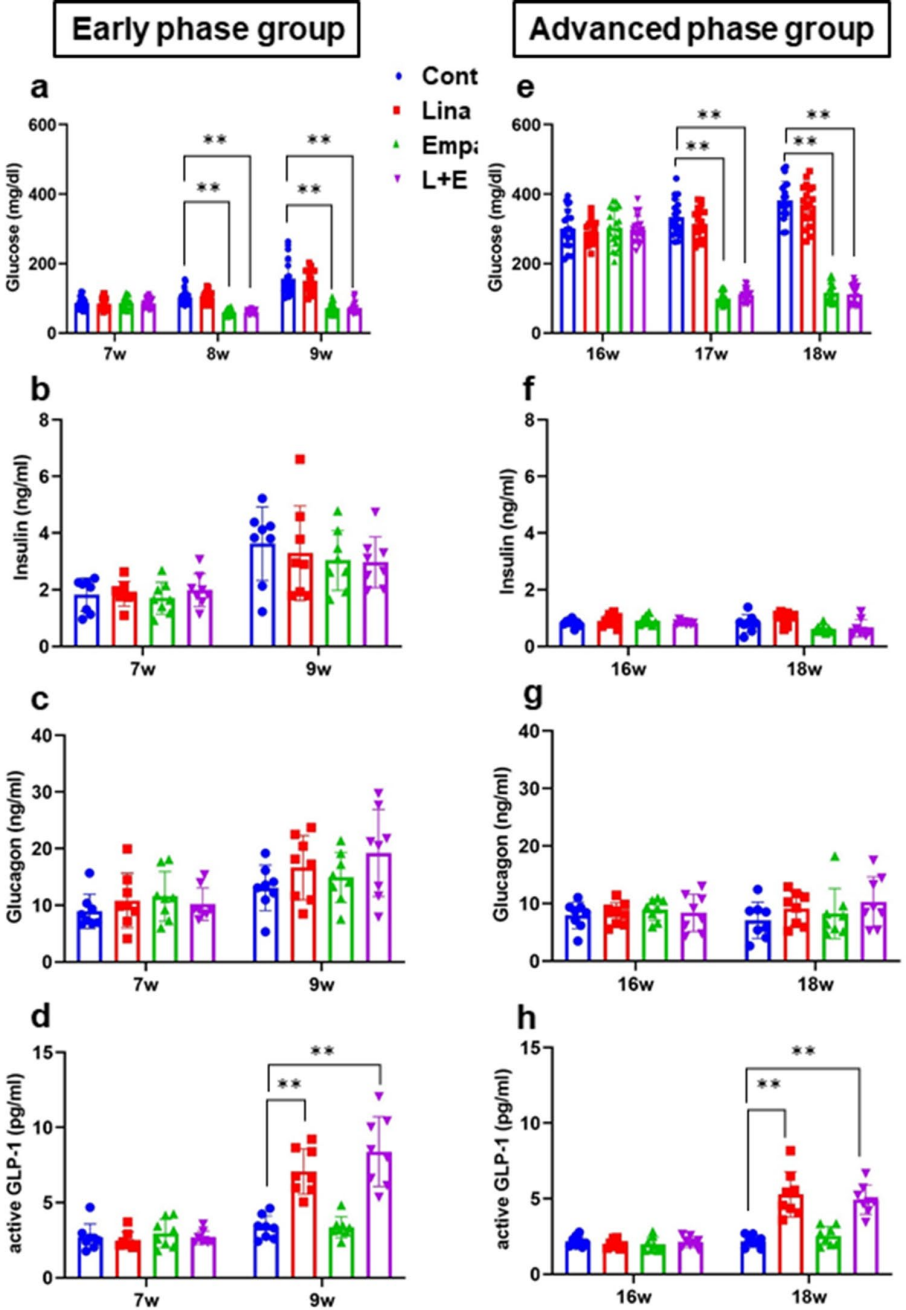
*db/db* mice in an early (7–9 weeks) and advanced (16–18 weeks) phase of diabetes treated with 0.5% carboxymethylcellulose (Cont: blue circle), linagligtin (Lina: red square), empagliflozin (Empa: green triangle), and combination of linagliptin + empagliflozin (L + E: purple reverse triangle) for 2 weeks. (**a**,**e**) Blood glucose levels (n = 20), (**b**,**f**) serum insulin levels (n = 8), (**c**,**g**) glucagon levels (n = 8), (**d**,**h**) active GLP-1 levels (n = 8). The multiple comparison was performed using the Tukey–Kramer method. Data are presented as mean ± S.D. **: *p* < 0.01.

After 2-week treatment, there was no difference in body weight and food intake among all groups in both an early and advanced phase of diabetes (Supplementary Fig. 1a–d). Empagliflozin and combination therapy significantly reduced blood glucose levels both in an early phase and an advanced phase of diabetes (Fig. 1a,e). Linagliptin and combination therapy significantly increased active GLP-1 levels, which suggested linagliptin was active although it did not change blood glucose levels (Fig. 1a,d,e,h). Active GIP levels were also significantly increased in linagliptin and combination therapy groups (Supplementary Fig. 1c,f). On the other hand, linagliptin, empagliflozin and combination therapy did not alter serum insulin and glucagon levels in both an early and advanced phase of diabetes (Fig. 1b,c,f,g). This result suggested that glucose-lowering effect of empagliflozin and combination therapy was mainly attributed to urinary glucose excretion but not insulin action. In addition, there were no significant differences in serum non-esterified fatty acid (NEFA), triglyceride (TG) and total cholesterol (TC) among the four groups in an early and advanced phase of diabetes (Supplementary Fig. 2a–f).

### Combination therapy exerted beneficial effects on β-cell function in an early phase but not in an advanced phase of diabetes

**Figure 2:**
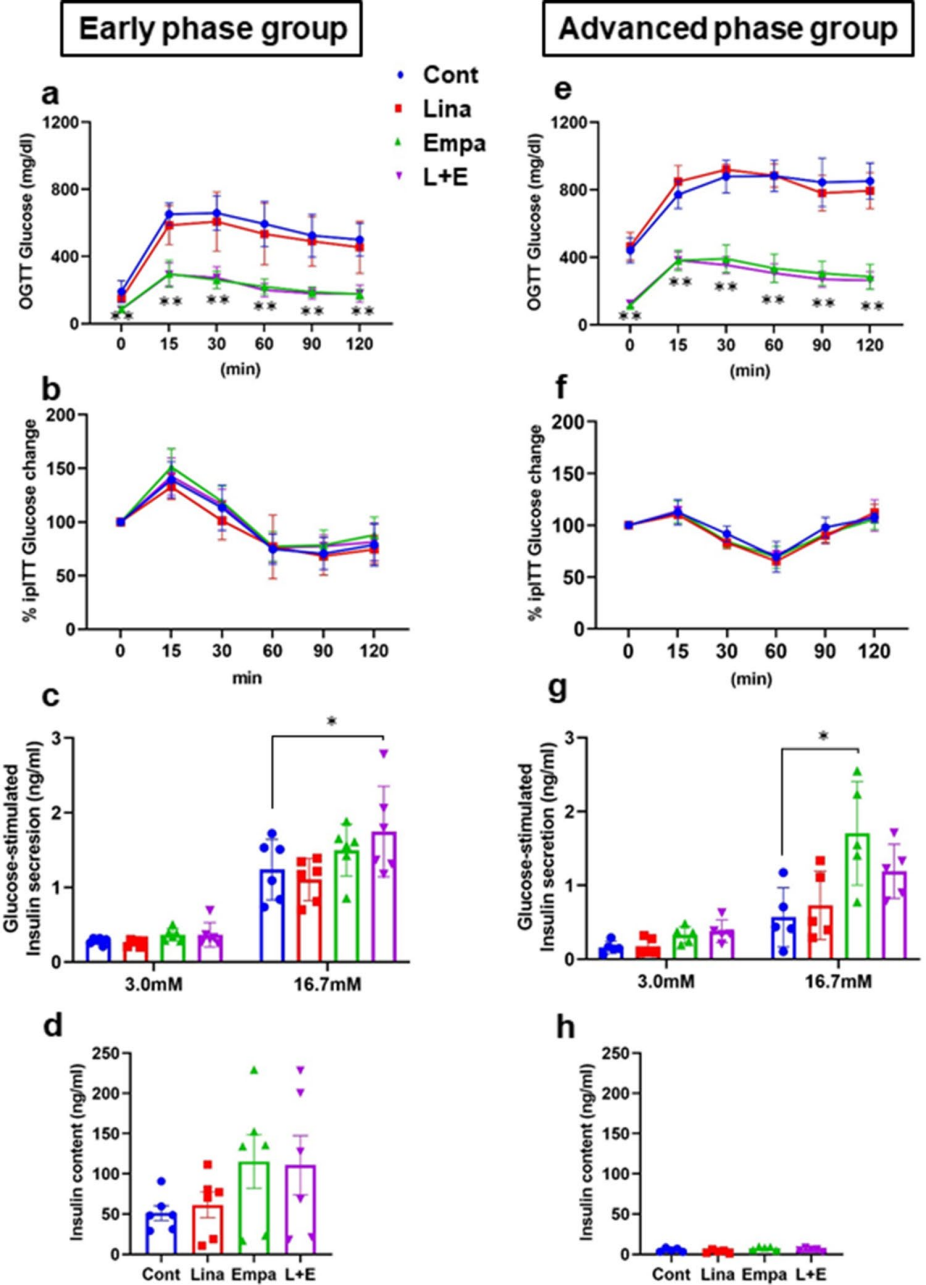
(**a**,**b**) Blood glucose levels after oral glucose tolerance test in an early and advanced phase of diabetes. (**b**,**f**) Blood glucose levels after insulin tolerance test in an early and advanced phase. (**c**,**g**) Glucose-stimulated insulin secretion (GSIS) was accessed after exposure to low (3.0 mmol/l) and high (16.7 mmol/l) glucose concentrations. (**d**,**h**) Insulin contents in pancreatic islets after GSIS. (**a**,**b**,**e**,**f**) n = 8, (**c**,**d**) n = 6, (**g**,**h**) n = 5. The multiple comparison was performed using the Tukey–Kramer method. Data are presented as mean ± S.D. *: *p* < 0.05, **: *p* < 0.01.

To evaluate the efficacy of each drug on glucose tolerance, we conducted oral glucose tolerance test (OGTT) one week after starting treatment. Glucose tolerance was significantly improved in empagliflozin and combination therapy groups, while linagliptin failed to improve glucose tolerance in both an early and advanced phase of diabetes (Fig. 2a,e, Supplementary Fig. 3a,d). Also, to evaluate the effects of these drugs on insulin resistance, we performed insulin tolerance test (ITT). As shown in Fig. 2b,f, Supplementary Fig. 3b,e, there was no difference among the 4 groups in an early and advanced phase. Furthermore, to evaluate the effect of these drugs on pancreatic β-cell function, we performed glucose-stimulated insulin secretion (GSIS). As shown in Fig. 2c,g, GSIS was improved in combination therapy in an early phase and empagliflozin in an advanced phase. Insulin content tended to be increased in empagliflozin and combination therapy in an early phase, although it did not reach a statistical significance (Fig. 2d,h). Taken together, 2-week treatment with empagliflozin and linagliptin improved β-cell function in diabetic *db/db* mice.

### Combination therapy increased β-cell mass only in an early phase but not in an advanced phase of diabetes

**Figure 3:**
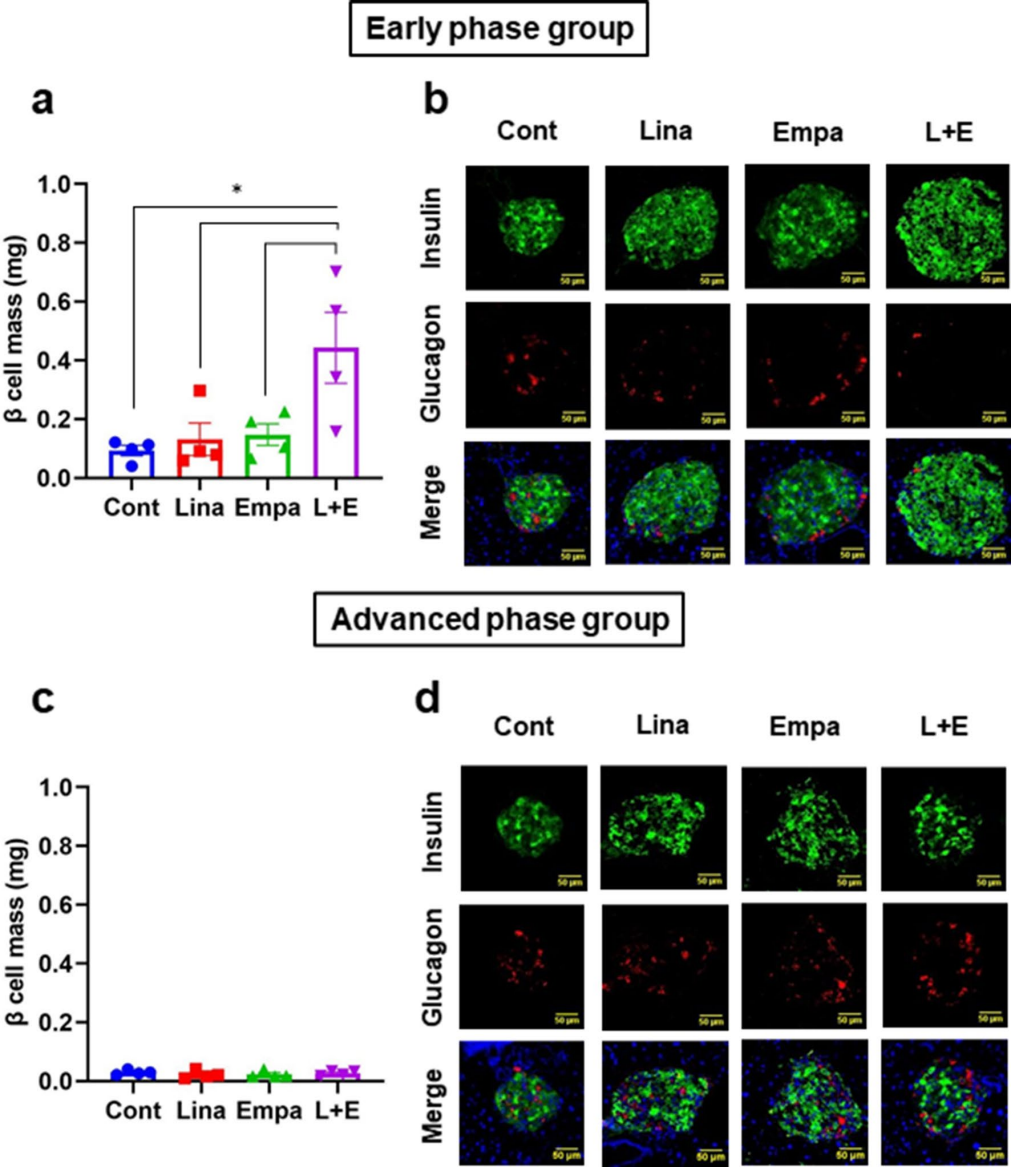
Pancreatic β-cell mass after 2-week treatment in (**a**) an early phase and (**b**) an advanced phase of diabetes (n = 4). (**b**,**d**) Representative immunofluorescent images of insulin (green) and glucagon (red) in pancreatic islet tissue sections. Scale bars: 50 μm. The multiple comparison was performed using the Tukey–Kramer method. Data are presented as mean ± S.D. *: *p* < 0.05.

First, to evaluate the effects of empagliflozin and/or linagliptin on β-cell mass, we conducted insulin-glucagon double staining. As shown in Fig. 3a,b, β-cell mass was significantly preserved only in combination therapy in an early phase of diabetes. On the contrary, β-cell mass in an advanced phase was drastically reduced in all groups compared to an early phase (Fig. 3c,d). In addition, β-cell mass preservation was not observed in all groups in advanced phase (Fig. 3c,d).

**Figure 4:**
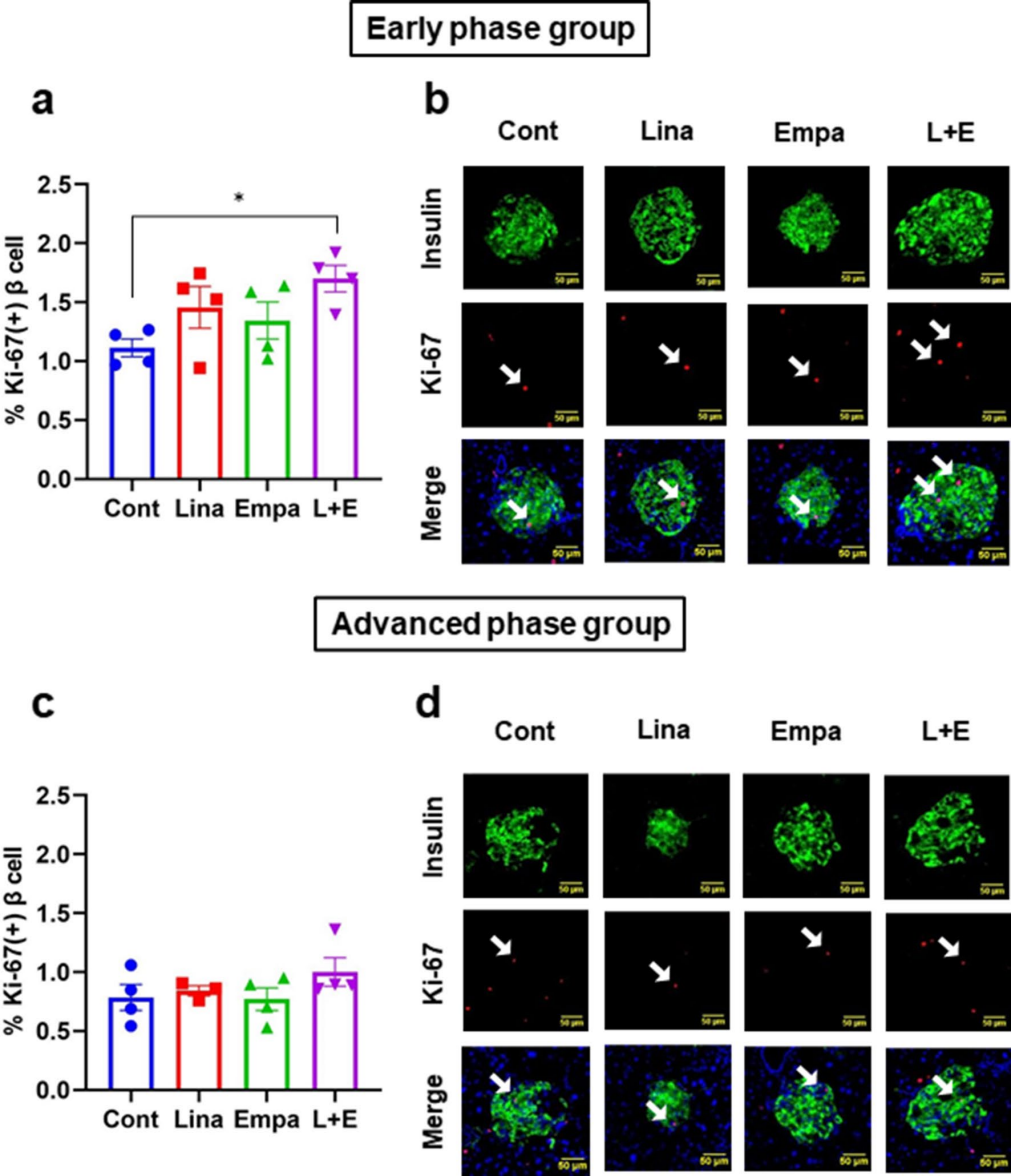
Ki-67 immunostaining in (**a**,**b**) an early phase and (**c**,**d**) an advanced phase of diabetes. (**a**,**c**) Quantification of Ki-67-positive β-cells (n = 4). (**b**,**d**) Representative images of Ki-67-positive β-cells. Scale bars: 50 μm. The multiple comparison was performed using the Tukey–Kramer method. Data are presented as mean ± S.D. *: *p* < 0.05.

Second, to examine the effects of these drugs on β-cell proliferation, we performed Ki67 staining. As shown in Fig. 4a,b, Ki67-positive β-cell ratio was significantly increased only in combination therapy in an early phase of diabetes, while no difference was observed in an advanced phase of diabetes (Fig. 4c,d).

**Figure 5:**
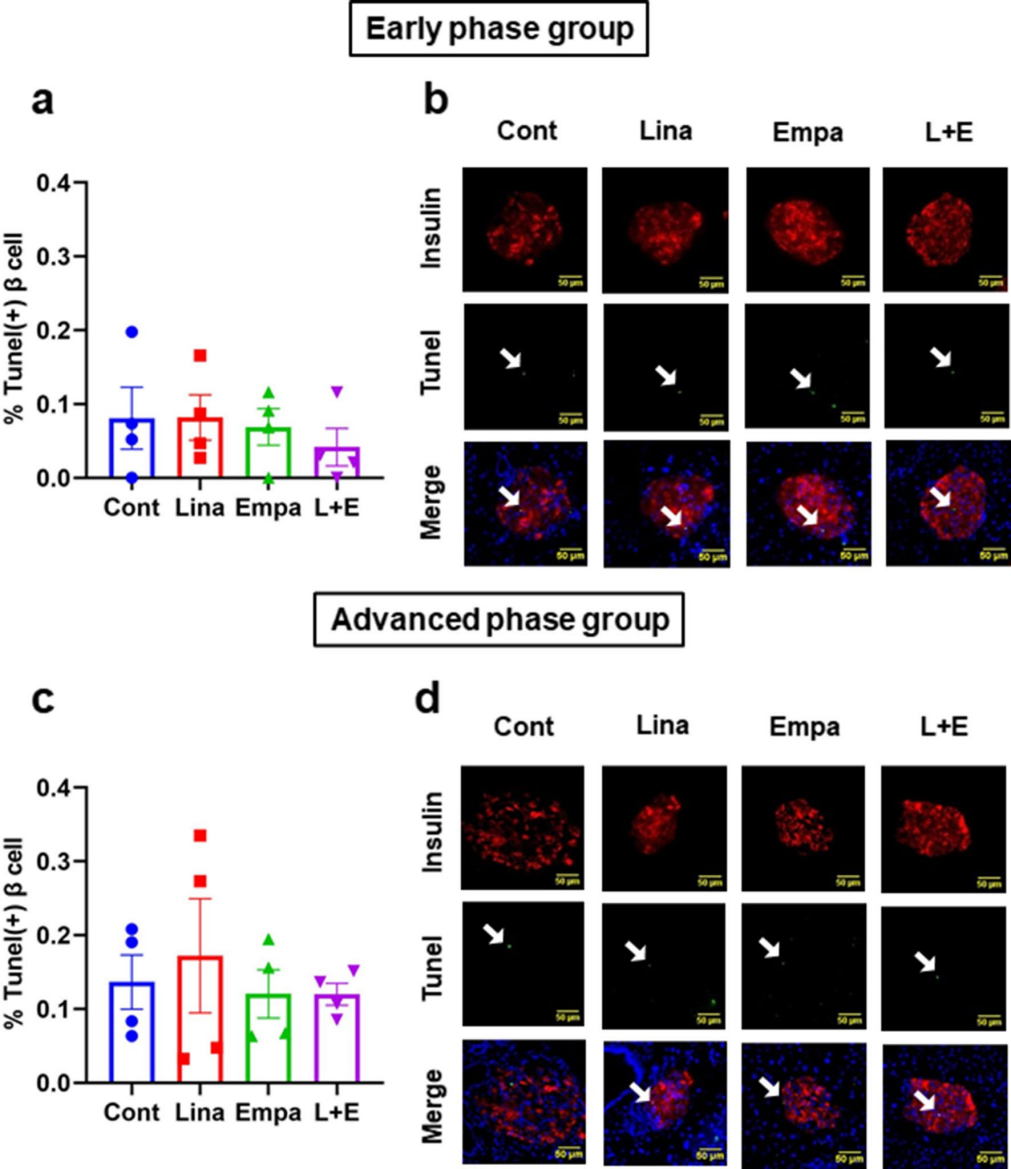
TUNEL assay in (**a**,**b**) an early phase and (**c**,**d**) an advanced phase of diabetes. (**a**,**c**) Quantification of β-cell apoptosis (n = 4). Scale bars: 50 μm. (**c**,**d**) Representative images of TUNEL positive β-cells. The multiple comparison was performed using the Tukey–Kramer method. Data are presented as mean ± S.D. *: *p* < 0.05.

Third, to examine the effects of these drugs on β-cell apoptosis, we performed TUNEL assay. There was no difference in TUNEL-positive β-cell ratio among all groups in both an early and advanced phase of diabetes (Fig. 5a–d). Taken together, β-cell mass was preserved in combination therapy, at least in part, due to increase of β-cell proliferation rather than decrease of apoptosis. We also evaluated fibrosis of β-cells by azan staining and mRNA expression levels related to fibrosis such as *Collagen1*, *Collagen4* and *Fibronectin*. There were no differences in such parameters among all groups in both an early and advanced phase of diabetes (Supplementary Fig. 4a–h).

### Various gene expression levels related to β-cells were significantly higher only in combination therapy in an early phase of diabetes

**Figure 6:**
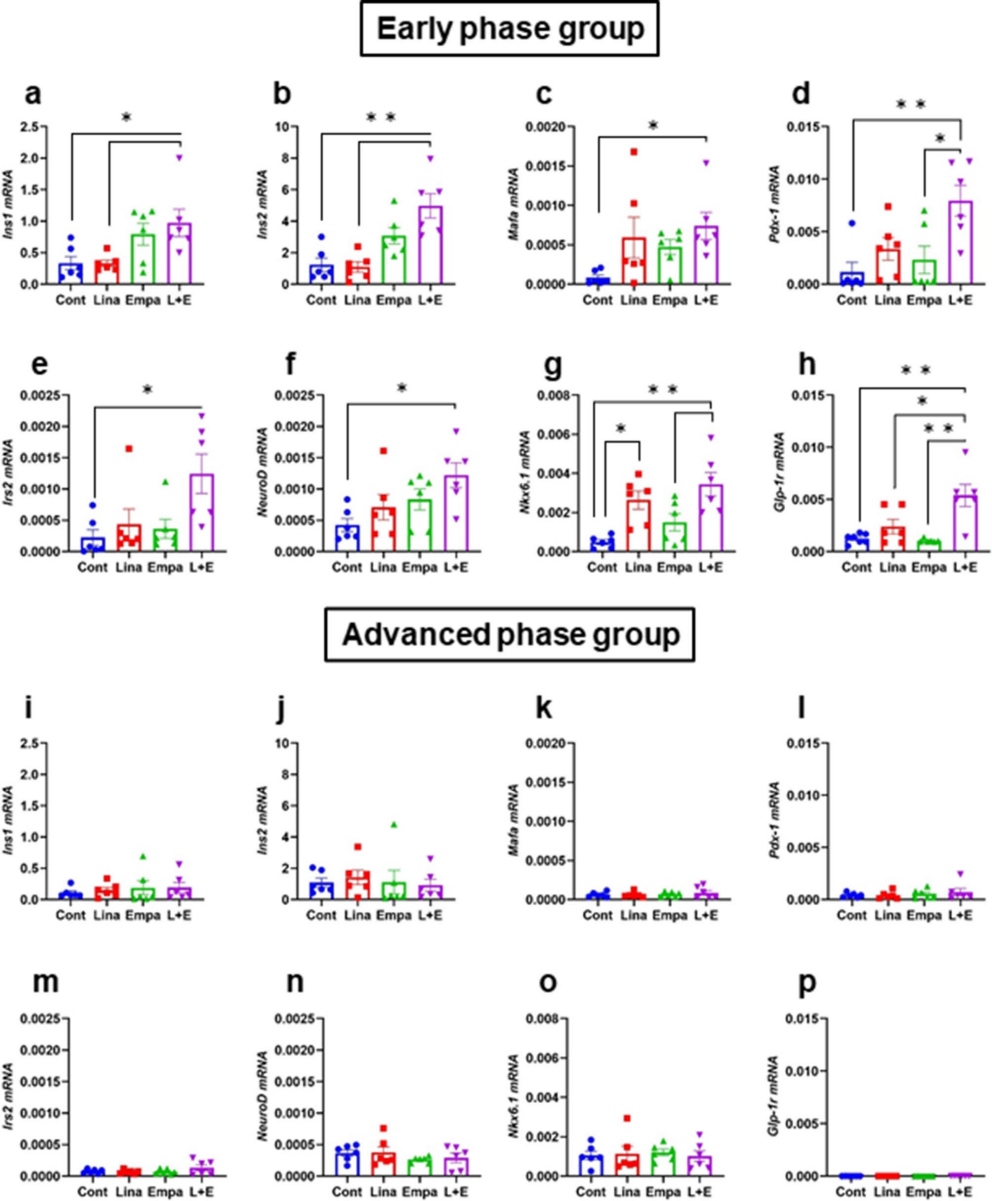
Expression levels of insulin and insulin gene transcription and GLP-1 receptor in (**a**–**h**) an early phase and (**i**–**p**) an advanced phase of diabetes. (**a**,**i**) *Insulin1*, (**b**,**j**) *Insulin2*, (**c**,**k**) *MafA*, (**d**,**l**) *Pdx-1*, (**e**,**m**) *Irs2*, (**f**,**n**) *NeuroD*, (**g**,**o**) *Nkx6.1*, (**h**,**p**) *Glp-1r* (n = 6). The multiple comparison was performed using the Tukey–Kramer method. Data are presented as mean ± S.D. *: *p* < 0.05.

We next investigated mRNA expression levels in isolated islets. mRNA expression levels of *Ins1*, *Ins2* and their transcription factors such as *Pdx-1* and *MafA* were all significantly increased in combination therapy (Fig. 6a–d). Immunohistological staining of PDX-1 and MafA confirmed that these important factors were increased in an early phase group of combination therapy not only in mRNA expression levels, but also in the protein levels (Supplementary Fig. 5a,b, 6a,b). In addition, mRNA expression levels related to β-cell differentiation and proliferation such as *NeuroD*, *Nkx6.1* and *Irs2* were significantly higher only in combination therapy in an early phase of diabetes (Fig. 6e–g). Importantly, mRNA expression level of *Glp-1r* was significantly higher in combination therapy in an early phase of diabetes (Fig. 6h), although there was no significant difference in *Gip-r* expression level (Supplementary Fig. 8i). These mRNA expression levels in an advanced phase of diabetes were all lower compared to an early phase of diabetes and comparable among all groups (Fig. 6i–p, Supplementary Fig. 8r). In addition, immunohistological staining of GLP-1R also presented higher GLP-1R expression in the protein level in combination therapy in an early phase of diabetes and its protein expression levels were quite similar to mRNA expression levels in all groups (Supplementary Fig. 7a,b). We also evaluated mRNA expression levels related to oxidative stress such as *Jnk* and *c-Jun*, ER stress such as *Bip* and *Chop*, inflammatory cytokines such as *Tnf-α*, *Il-6* and *Il-1β*, and *Bcl2*, which is an important molecule in GLP-1 signaling. There were no differences in all expression levels among the 4 groups in early and advanced phase of diabetes (Supplementary Fig. 8a–r).

### Two-week treatment with each drug and/or combination have little effect on the liver and epidydimal white adipose tissue as a whole

We next investigated the effects of linagliptin, empagliflozin and combination therapy on the liver and epidydimal white adipose tissue. In HE staining of the liver, there was no obvious difference among all 4 groups. Furthermore, liver TG contents were comparable among all 4 groups in both an early phase and an advanced phase (Supplementary Fig. 9a–d). In early phase group, mRNA expression level of *Srebp1c* was significantly lower in drug treated groups compared to control group, while such significancy was diminished in an advanced phase (Supplementary Fig. 10a,k). However, other lipogenic factors such as *Fas* and *Scd1* expression levels were comparable among all 4 groups (Supplementary Fig. 10b,c,l,m). mRNA expression levels related to β-oxidation such as *Cpt1α* and *Pparα* were comparable among all 4 groups in both an early phase and an advanced phase (Supplementary Fig. 10d,e,n,o). In an early phase, *Pepck* expression level was significantly increased compared to linagliptin and combination therapy, while there was no difference in *G6pase* expression level among all 4 groups (Supplementary Fig. 10f,g). These results suggest substantial hepatic glucose production might be increased in empagliflozin treated group in an early phase. On the contrary, *Pepck* expression level was not increased in an advanced phase (Supplementary Fig. 10p,q). mRNA expression levels of inflammatory markers such as *Tnfα, Il-6 and Mcp1* were comparable in all 4 groups in both an early phase and an advanced phase (Supplementary Fig. 10h,i,j,r,s,t). In epidydimal WAT, there was no significant differences in HE staining and adipocyte size among all 4 groups in both an early and an advanced phase (Supplementary Fig. 11a–d). Various mRNA expression levels related to lipogenesis, inflammatory markers, macrophage markers and adiponectin were all comparable among all 4 groups in both an early phase and an advanced phase (Supplementary Fig. 12a–r). These results suggest that 2-week treatment have little effect on the liver and white adipose tissue as a whole.

## Discussion

In this study, we demonstrated combination therapy of empagliflozin and linagliptin exerts more beneficial effects on β-cell mass and function in an early phase of diabetes. Interestingly, there was no change in villain factors on β-cells such as oxidative stress, ER stress and inflammatory cytokines (Supplementary Fig. 8a–g,j–p). In this study, *Glp-1r* expression level was preserved only in combination therapy. Therefore, it is possible that preserved *Glp-1r* and increased serum active GLP-1 exerted β-cell protective effects through GLP-1 signaling in β-cells without improvement of villain factors such as oxidative stress, ER stress and inflammatory cytokines. It is well known that insulin signaling is important for the maintenance of pancreatic β-cell mass and function^23–26^. However, as there was no change in serum insulin levels among all groups, we assume that direct insulin pathway via insulin receptor has little impact on β-cell mass and function in this study (Fig. 1b,f).

It is reported that DPP-4 inhibitors improve β-cell mass and function by via β-cell differentiation and proliferation accompanied by decrease of oxidative stress, ER stress and inflammatory cytokines^8,27–29^. It is also reported that SGLT2 inhibitors improve β-cell mass and function via similar mechanisms as DPP-4 inhibitors, although the mechanism of glucose-lowering effect is totally different^9,10,30^. On the contrary, in our study, two-week administration of either linagliptin or empagliflozin failed to improve β-cell mass and function (Fig. 3a,c). The discrepancy of the result might be attributed to short-term intervention, which was 2 weeks in this study, while it was at least longer than 4 weeks in previous reports. In addition, the difference of drug administration methods such as oral gavage or mixed in the meal might have strongly affected the results as it is known that drug turnover such as metabolism and excretion is quite fast especially in rodents. Importantly, it has been reported that under diabetic conditions GLP-1 receptor expression in β-cells is down-regulated by gluco-lipotoxicity^31–35^. We previously reported mRNA expression level of *Glp-1r* in isolated islets was increased after two-week treatment in *db/db* mice^10^. In this study, however, *Glp-1r* expression level in isolated islets was not recovered after administration of empagliflozin (Fig. 6h,p), although blood glucose level was significantly decreased (Fig. 1a,e). As the reason, we cannot completely deny the influence of difference of drug and its delivery method between our previous report and this study. As shown in Supplementary Table 1, drugs were administered by mixed in the meal or water in studies using SGLT2 inhibitors in previous reports^9,10,30,36^. As *db/db* mice constantly eat and drink through dark cycle and light cycle, it is possible that drug concentration is maintained compared to oral gavage administration. This could cause large differences in phenotype.

In contrast, active GIP levels were higher in linagliptin and combination therapy. However, there was no difference in mRNA expression levels of *Gip-r* among all groups. It has been reported that the *Gip-r* expression is downregulated by hyperglycemia^33,37–39^. Piteau et al. beautifully presented that phlorizin administration recovered *Gip-r* mRNA expression levels in pancreatic β cells in ZDF rats^40^. However, in our study, such recovery was not observed. This might be derived from the differences of treatment term and species of animal used in the studies. On the contrary, Shimo N et al. reported mRNA expression level of *Gip-r* was not recovered in islets isolated from *db/db* mice treated with 10 mg/kg empagliflozin for one week^41^. Taken together, it might need longer treatment to observe recovery of mRNA expression level of *Gip-r* in *db/db* mice.

In this study, combination therapy significantly increased β-cell mass and improved β-cell function in an early phase of diabetes. In mRNA expression levels in isolated islets, insulin and its transcriptional factors such as *Pdx-1* and *MafA* were significantly increased in combination therapy in an early phase of diabetes (Fig. 6c,d). Immunohistological study confirmed that PDX-1 and MafA were actually increased in combination therapy in an early phase in the protein level (Supplementary Fig. 5a,b, 6a,b). Therefore, we speculate that β-cell function would be more clearly ameliorated if intervention term is longer. In combination therapy, β-cell mass was increased approximately 4 folds compared to control group (Fig. 3a). In mRNA expression levels in isolated islets, increased *Glp-1r* and *Irs2* could, at least in part, explain the increase of β-cell mass (Fig. 6e,h). However, we still cannot deny the involvement of re-differentiation of β-cells, α- to β-cell conversion and the influence of humoral factors as previously reported^42,43^. It still needs further investigation to clarify this point. In addition, although combination therapy exerted beneficial effects on both β-cell mass and function in an early phase of diabetes in this study, β-cell mass was increased more clearly and to a larger extent, compared to the extent of β-cell function improvement. Therefore, we assume that increase of β-cell mass precedes improvement of β-cell function after such treatment. We feel that this point is very important in islet biology research area and in therapeutic strategy for type 2 diabetes, although further study would be necessary to conclude this point.

Interestingly, *Glp-1r* expression level was increased only in combination therapy in an early phase of diabetes (Fig. 6h). This result suggests that reduction of glucose toxicity alone cannot lead to alteration of *Glp-1r* expression level. In fact, although empagliflozin indeed successfully ameliorated glucose toxicity, it failed to increase *Glp-1r* expression level. In addition, linagliptin fully exerted its effect when we observed an increase of active GLP-1 levels in linagliptin and combination therapy (Fig. 1d,h). Therefore, we assume that short-term exposure to GLP-1 receptor agonist under ameliorated glucose toxicity might play some important roles to increase *Glp-1r* expression level in isolated islets. It is still not clearly understood how *Glp-1r* expression is regulated in vivo. We think that our data raised interesting clues for future investigation.

Shimoda M et al. elucidated two-week treatment with liraglutide improved pancreatic β-cell mass and function in *db/db* mice^31^. In our study, two-week linagliptin treatment failed to improve pancreatic β-cell mass and function. This difference is probably derived from the difference of GLP-1 concentration as linagliptin can only preserve GLP-1 concentration within the physiological range, while liraglutide increase GLP-1 concentration beyond the physiological concentration. Moreover, several parameters such as body weight, blood glucose level and lipid profile are improved and other organs are not explored in this study. Hansen HH et al. reported that both 10 mg/kg empagliflozin and liraglutide preserved β-cell mass after 4 and 8 weeks of treatment, whereas only empagliflozin showed a reduced rate of β-cell mass loss after 8-week treatment in Zucker diabetic fatty rats^44^. In our study, two-week treatment with linagliptin or empagliflozin did not present such strong effect on β-cell mass. This might be derived from the intervention term. The reason why empagliflozin showed a reduced rate of β-cell mass loss compared to liraglutide might be due to down-regulation of GLP-1R in pancreatic β-cells after long term exposure to GLP-1R agonist of high concentration. Anyway, both studies did not explore *Glp-1r* expression level in pancreatic β-cells and other organs, it is hard to directly compare our study and these previous reports.

Recently, Gray S et al. reported that there is a discordance between GLP-1R gene and protein expression in mouse pancreatic islet cells^45^. They elucidated that over 90% of β-cells are GLP-1R positive. On the other hand, α-cells do not express mRNA of *Glp-1r* and δ-cells express mRNA of *Glp-1r*, but not GLP-1R protein. In addition, they also elucidated multiparous mice, which is considered metabolic stress model, presented decreased mRNA expression level of *Glp-1r* in pancreatic β-cells compared to nulliparous mice, but preserved GLP-1R protein and GSIS. This study demonstrated discrepancy between mRNA expression and protein level of GLP-1R in pancreatic β-cells. In our study, GLP-1R protein level was increased accompanied with increased mRNA expression of *Glp-1r* in combination therapy in an early phase of diabetes (Fig. 6h, Supplementary Fig. 7a). Multiparous mice present increased body weight with normoglycemia. Therefore, it still needs further investigation whether there is such discrepancy when mice are hyperglycemic such as *db/db* mice.

The limitation of our study is that we did not evaluate long-term treatment from 7 to 18 weeks of age. Although it is a matter of speculation, it is possible that a longer-term intervention with linagliptin and/or empagliflozin from an early phase of diabetes would exert more beneficial effects on pancreatic β-cell mass and function and would be appropriate to assess accumulative phenomenon such as fibrosis. We did not evaluate such accumulative phenomenon in this study. However, a very important point is that our goal in this study was to evaluate the effect of each drug and combination on pancreatic β-cell mass and function without affecting glucose levels for linagliptin to evaluate the direct effects of incretin on pancreatic β-cell and moreover without affecting other insulin sensitive organs such as the liver and adipose tissue and serum lipid profile. Therefore, we limited intervention term for two weeks in this study. As shown in Supplementary Table 1, various studies of DPP4 inhibitors and SGLT2 inhibitors used different animal models, drug dosage, drug delivery methods and intervention terms for evaluation of pancreatic β-cell mass and function^8-10,28,30,36^. For the evaluation of DPP4 inhibitors, animals such as KKAy and ICR treated with streptozotocin and HFD to induce mild diabetes were used as DPP4 inhibitors improves glucose tolerance and insulin sensitivity in these mild diabetes models. However, DPP4 inhibitors do not improve glucose metabolism in *db/db* mice^46^. In addition, most studies which evaluate drug effectiveness on pancreatic β-cell do not assess other insulin sensitive organs such as the liver and adipose tissue, which would strongly influence pancreatic β-cell function indirectly. In the aspect of these fact, our study demonstrated the direct incretin effects on pancreatic β-cell without improvement of blood glucose and alternation of major phenotype of the liver and adipose tissue.

In conclusion, combination therapy of DPP-4 inhibitor linagliptin and SGLT2 inhibitor empagliflozin increased β-cell mass and enhanced β-cell function in an early phase of diabetes in diabetic *db/db* mice. The data in this study clearly suggest that it would be favorable to start combination therapy of DPP-4 inhibitor and SGLT2 inhibitor in an early phase of diabetes in order to efficiently preserve β-cell mass and function.

## Methods

### Animals and diet

We used 7-week-old male BKS.Cg- + Lepr db/ + Lepr db/Jcl (*db/db*) mice as an early phase of diabetes and 16-week-old male *db/db* mice as an advanced phase of diabetes as previously reported^10^. Animals were purchased from Clea, Tokyo, Japan. They were housed two animals per cage in all experiments under controlled ambient conditions and a 12:12 h light/dark cycle. Animals were given free access to water and standard chow (MF; Oriental Yeast CO., LTD., Japan) and were maintained at 25 °C. These were divided into 4 groups, and each group was treated with linagliptin (Lina: 3 mg/kg, daily oral gavage), empagliflozin (Empa: 30 mg/kg, daily oral gavage), linagliptin and empagliflozin combination (L + E), or control (Cont: 0.05% carboxymethylcellulose, daily oral gavage) for 2 weeks^47^. Body weight and food intake were monitored weekly. All methods were carried out in accordance with relevant guidelines and regulations. This study was approved by the Animal Use Committee of Kawasaki Medical School (No.18–090) and was conducted in accordance with the Animal Use Guidelines of the Kawasaki Medical School. We confirmed that this study was carried out in compliance with the ARRIVE guidelines.

### Measurement of biochemical markers

For measurements of active glucagon-like peptide-1 (GLP-1), active glucose-dependent insulinotropic polypeptide (GIP) and glucagon, EDTA and aprotinin were placed in sample collection tubes beforehand. Active GLP-1 level was measured using GLP-1, active form assay (IBL, Gunma, Japan). Active GIP level was measured using mouse GIP (active) ELISA Kit (YK252, Yanaihara Institute Inc. Shizuoka, Japan). Glucagon level was determined using glucagon ELISA (Mercodia, Uppsala, Sweden). Serum insulin level was determined using a mouse insulin ELISA kit (Morinaga, Tokyo, Japan). Serum total cholesterol, free fatty acid and serum triglyceride levels were also assayed using enzymatic methods (FUJIFILM Wako Pure Chemical Corporation, Osaka, Japan) in accordance with the manufacturer’s instructions. Measurements were conducted in accordance with previous reports^13,48^.

### Oral glucose tolerance test (OGTT)

After 16 h fasting, D-( +)-glucose (1 g/kg BW) was administered orally for OGTT. Blood samples from tail snips were collected at the indicated time point and blood glucose levels were measured using Glutest Mint (Sanwa Kagaku Kenkyusho, Mie, Japan). The experiment was conducted in accordance with the previous report^9,13,48^.

### Insulin tolerance test (ITT)

Insulin tolerance test was performed by intraperitoneal injection (2 U/kg BW) of human regular insulin (Novo Nordisk, Bagsvaerd, Denmark) after a 5-h fast at 8 weeks of age for an early phase and 17 weeks of age for an advanced phase of diabetes. Blood glucose levels were monitored at 0, 15, 30, 60, 90 and 120 min after insulin injection as previously reported^9^.

### Liver TG contents

Liver TG contents were measured as previously reported^49^.

### Pancreatic islet isolation

Isolation of islets from the pancreas of the mice was conducted as previously described^48^. In brief, after ligation of the common bile duct with silk thread at a point close to the duodenal outlet, 2.5 ml of Hanks’ Balanced Salt Solution (HBSS) (Sigma, St Louis, MO, USA) containing 0.6 mg Liberase TL (Roche Diagnostics, Tokyo, Japan) and 25 mmol/l HEPES were injected into the duct. The swollen pancreas was removed and incubated at 37 °C for 24 min. The pancreatic tissue was then dispersed by pipetting and washed twice with ice-cold HBSS containing 25 mmol/l HEPES and 10% (wt/vol.) FBS. Thereafter, the islets were manually picked up under a stereoscopic microscope and used immediately for the experiments.

### Glucose-stimulated insulin secretion (GSIS) with isolated pancreatic islets

To examine GSIS, size-matched pancreatic islets were prepared (five pancreatic islets/tube) and pre-incubated in KRB–HEPES buffer. The supernatant fraction was replaced with glucose solution (either 3 mmol/l or 16.7 mmol/l) and the mixture was incubated for an additional 60 min at 37 °C as previously reported^9,10,48^. The supernatant fraction and islets were stored at − 80 °C until use.

### Histological and immunohistochemical analyses of islets

Isolated pancreases were fixed overnight with formaldehyde at 4 °C. Tissues were routinely processed for paraffin embedding and 4 μm sections were cut and mounted on silanised slides. Insulin-glucagon double staining and Ki-67 staining were conducted as previously reported^9,10,48^. To investigate cell apoptosis in pancreatic islets, a TUNEL assay was performed using a DeadEnd Fluorometric TUNEL System (DeadEnd; cat. no. G3250, Promega, Madison, WI, USA) as previously reported^9,10,48^. For immunostaining of MafA, PDX-1 and GLP-1R, the following antibodies were used as a primary antibody (MafA: ab264418 (1:500), PDX-1: CST 5679 (1:100), GLP-1R: DSHB Hybridoma Product Mab 7F38 (1:15.5). As secondary antibodies, donkey anti-rabbit IgG (H + L) secondary antibody, Alexa Fluor 594 conjugate (Life Technologies, A-21207 (1:1000)) for MafA, donkey anti-rabbit IgG (H + L) secondary antibody, Alexa Fluor 488 conjugate (Invitrogen, A-21206 (1:1000)) for PDX-1 and donkey anti-mouse IgG (H + L) secondary antibody, Alexa Fluor 594 (Life Technologies, A-21203 (1:1000)) for GLP-1R were used.

### Morphometric analysis

The image analysis software NIH Image (version 1.61; http://rsbweb.nih.gov/ij/) was used to calculate the pancreas area and islet area. Using a total of nine sections (three sections from three different areas of the pancreas) for each group of mice, β-cell mass was estimated via the following formula: cell mass (mg) = average of islet area per section/average of pancreas area per section × weight of pancreas × β-cell ratio (average of β-cell number/cell number in islet). Observations were made using a minimum of 50 islets and, when quantified, were expressed as a percentage of the total number of islet cells as previously reported^9,10,48^.

All-in-One Fluorescence Microscope BZ-X800 analysis application (KEYENCE, Japan) was used for the measurement of adipocyte size according to the manufacture’s information.

### RNA preparation and quantitative PCR

Total RNA was extracted from isolated islets using an RNeasy mini kit (Qiagen, Valencia, CA, USA) according to the manufacturers’ instructions. cDNA was produced from mRNA using TaqMan reverse transcription reagents (Applied Biosystems, Foster City, CA, USA). Quantitative PCR (qPCR) was performed using a 7500 Real-Time PCR system (Applied Biosystems) as previously reported^48^. The relative expression levels were compared by normalization to the expression levels of 18sr. The primer sequences are shown in Supplementary Table 2.

### Statistics

Results are expressed as mean ± SD. Graph Pad Prism 8 was used for figure formation and statistical analysis. Differences among more than two groups were tested using the Tukey–Kramer method. *p* values less than 0.05 were considered to denote statistical significance.

## Supplementary Information

Supplementary Information.

## Data Availability

The datasets used and/or analyzed in the current study are available from the corresponding authors upon reasonable request.
