# Supplementary material for: Early combination therapy of empagliflozin and linagliptin exerts beneficial effects on pancreatic β cells in diabetic db/db mice
Source: Sci Rep. 2021 Aug 9;11:16120. doi: 10.1038/s41598-021-94896-w (PMC8352868; doi:10.1038/s41598-021-94896-w)
Supplement: Supplementary file 1 — Supplementary Information. [file 41598_2021_94896_MOESM1_ESM.pdf]

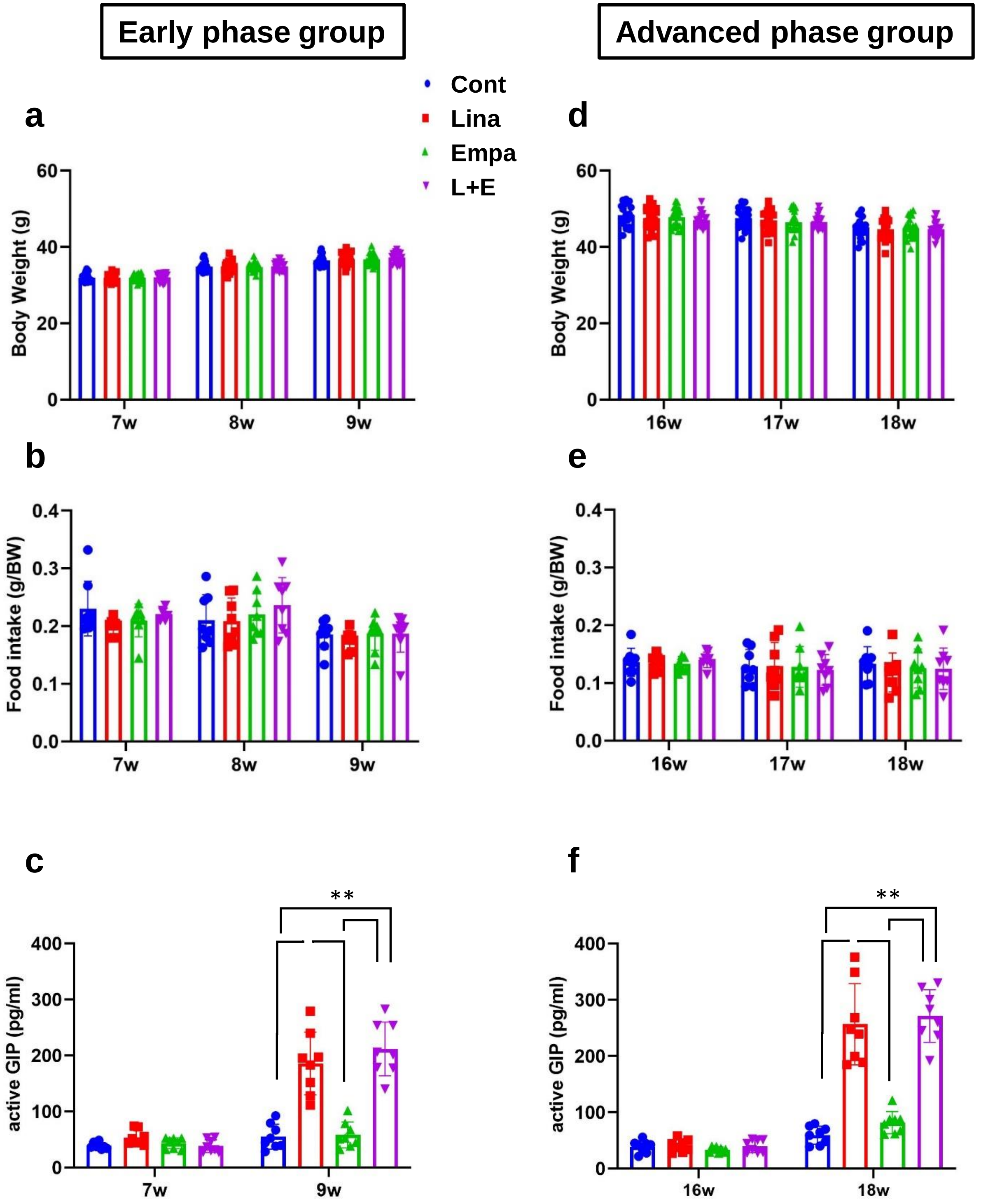

Supplementary Figure 1. Body weight and food intake in (a) (b) an early phase and (d) (e) an advanced phase of diabetes (n=20). Active GIP levels in (c) an early phase and (f) an advanced phase (n=8). The multiple comparison was performed using the Tukey-Kramer method. Data are presented as mean ± S.D. **: $p<0.05$.

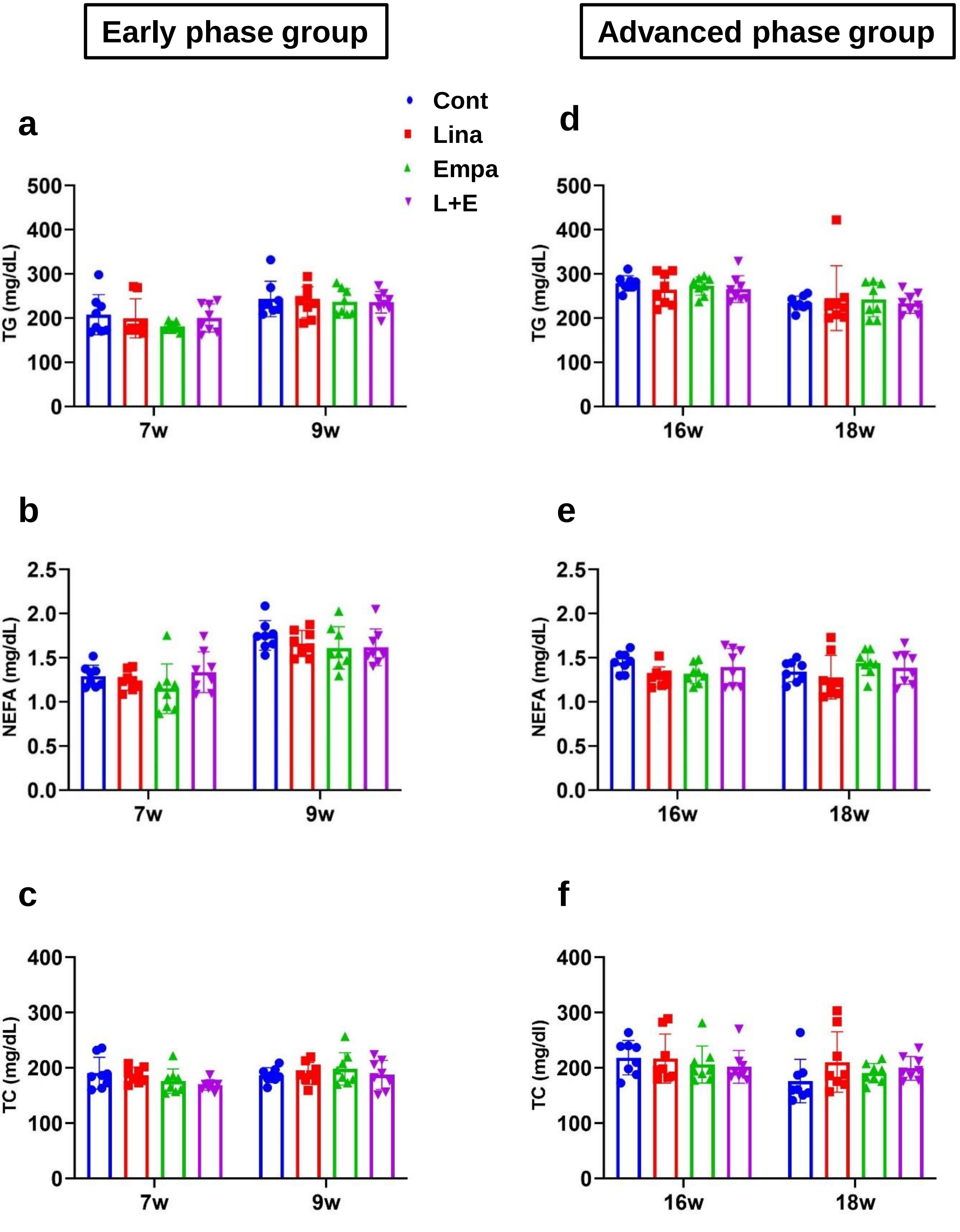

Supplementary Figure 2. Area under the curve of OGTT and ipITT in (a)(b) an early phase and (d)(e) an advanced phase of diabetes (n=6).

Early phase group **Advanced phase group**

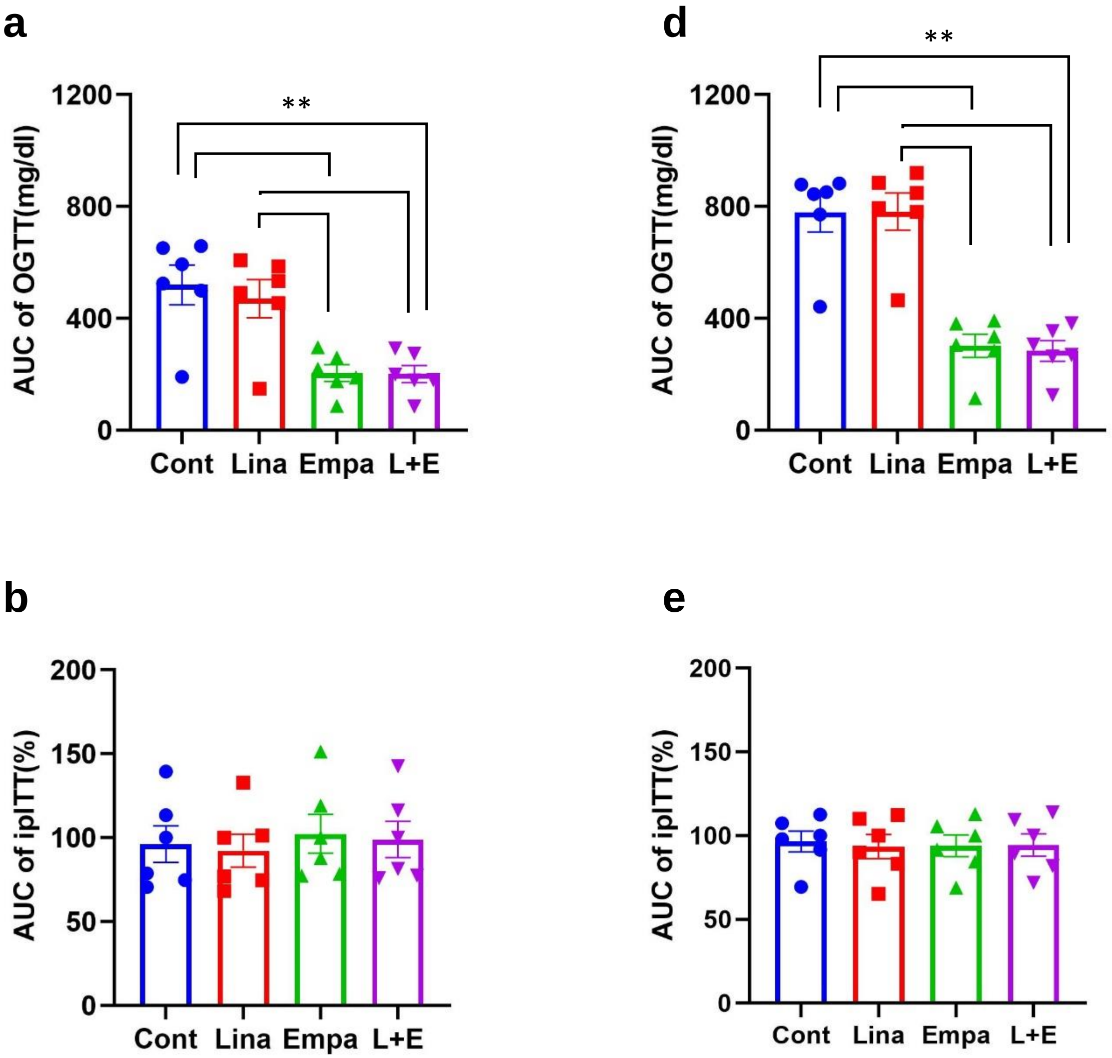

Supplementary Figure 3. Serum TG, NEFA and TC levels in (a-c) an early and (d-f) an advanced phase of diabetes. (n=8) The multiple comparison was performed using the Tukey-Kramer method. Data are presented as mean ± S.D.

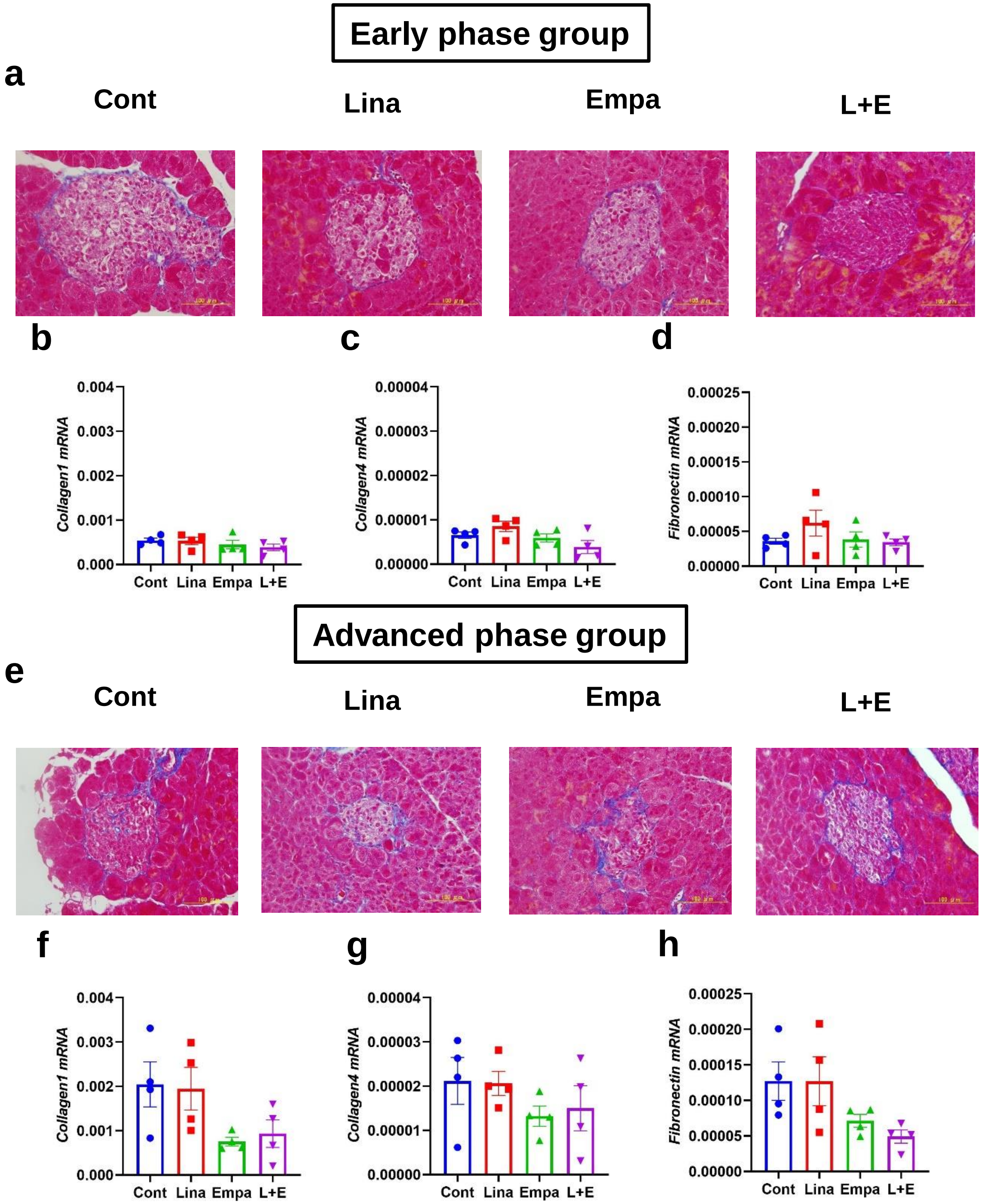

Supplementary Figure 4. Azan staining of pancreatic β-cells in (a-d) an early phase and (e-h) an advanced phase of diabetes. (a) (e) Representative images of Azan staining. Scale bars: 100 μm. (b-d) (f-h), mRNA expression levels related to fibrosis. (b) (d) *Collagen1*, (c) (g) *Collagen4*, (d) (h) *Fibronectin*. (n=4) The multiple comparison was performed using the Tukey-Kramer method. Data are presented as mean ± S.D.

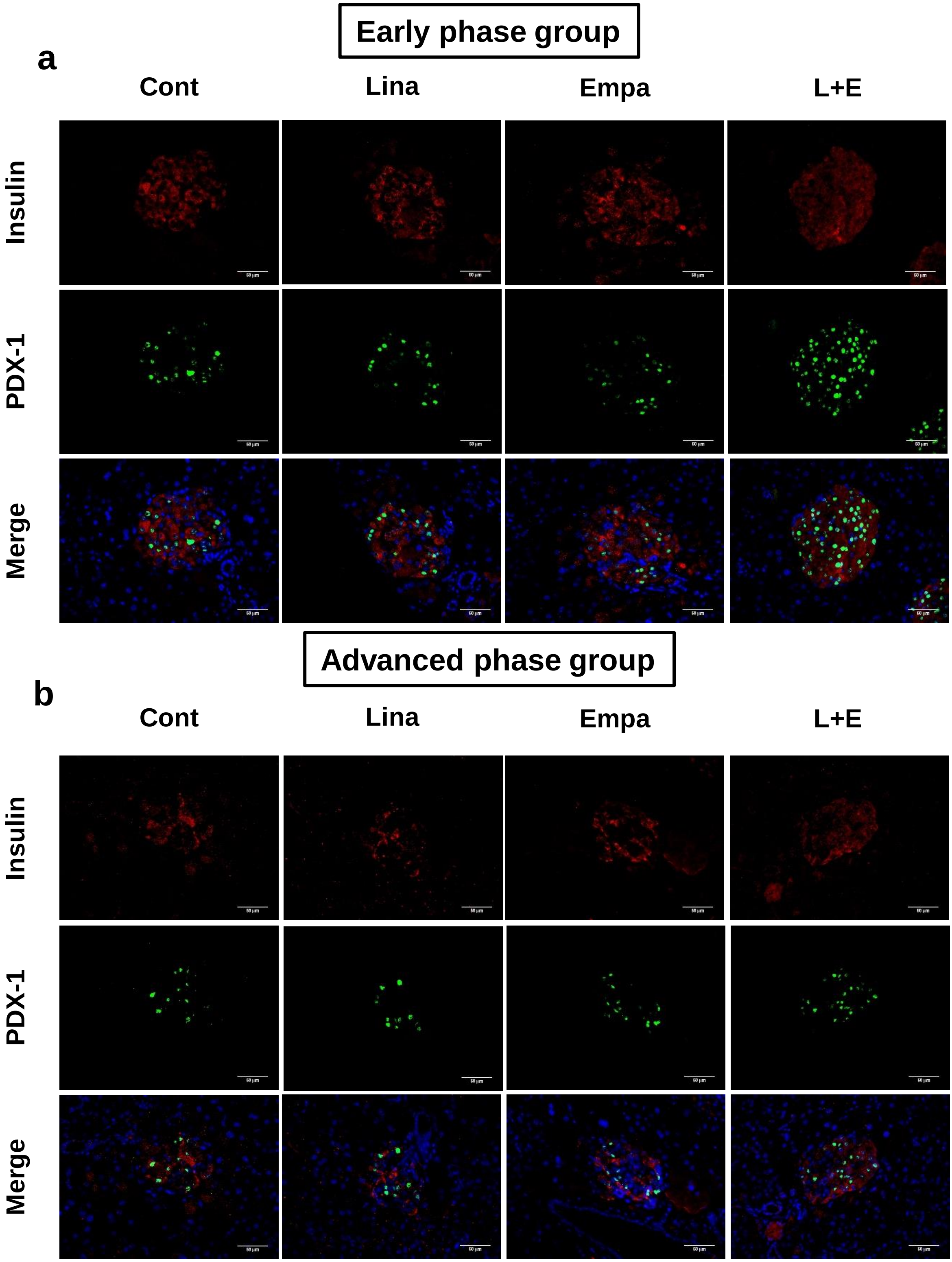

Supplementary Figure 5. Immunohistological staining of PDX-1 and insulin in (a) an early phase and (b) an advanced phase.

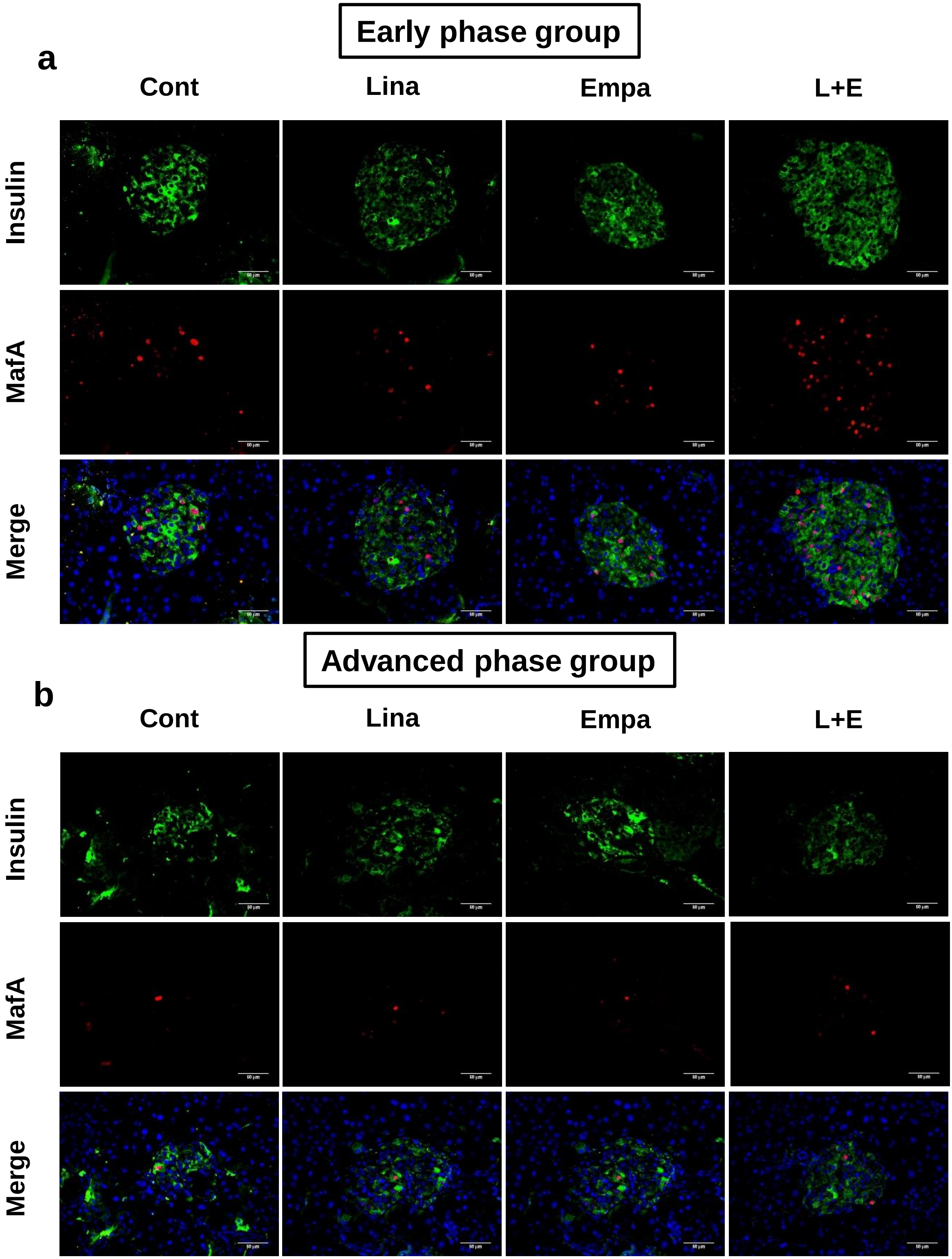

Supplementary Figure 6. Immunohistological staining of MafA and insulin in (a) an early phase and (b) an advanced phase.

Early phase group

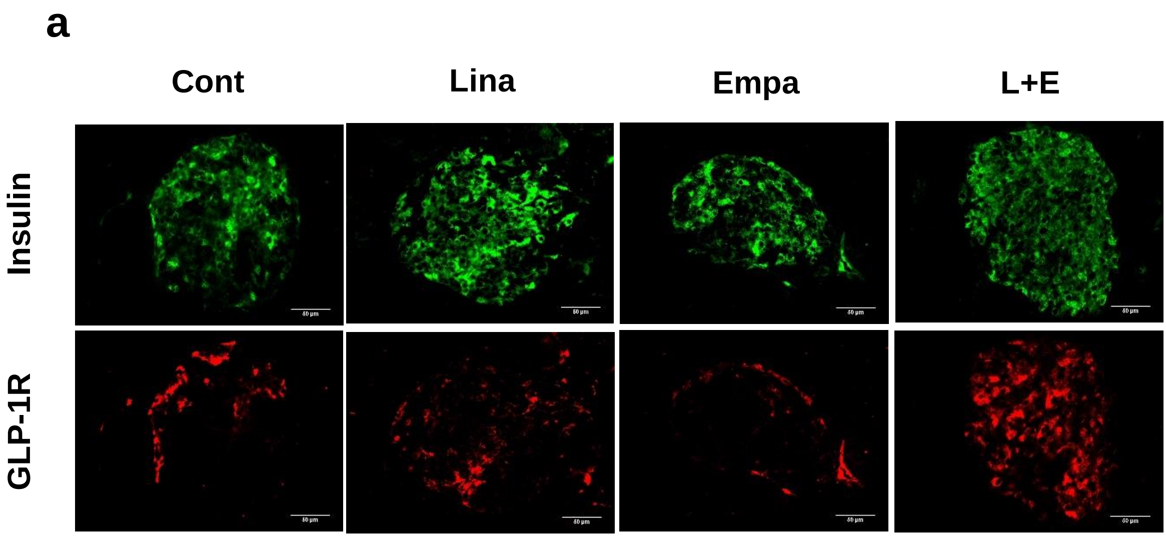

Advanced phase group

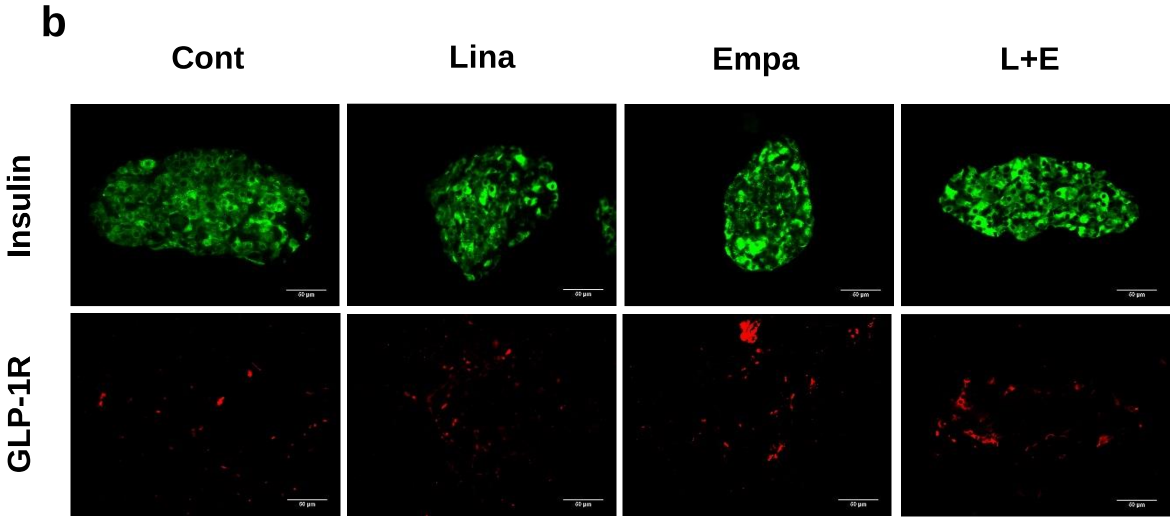

Supplementary Figure 7. Imuunohistological staining of GLP-1R and insulin in (a) an early phase and (b) an advanced phase.

Early phase group

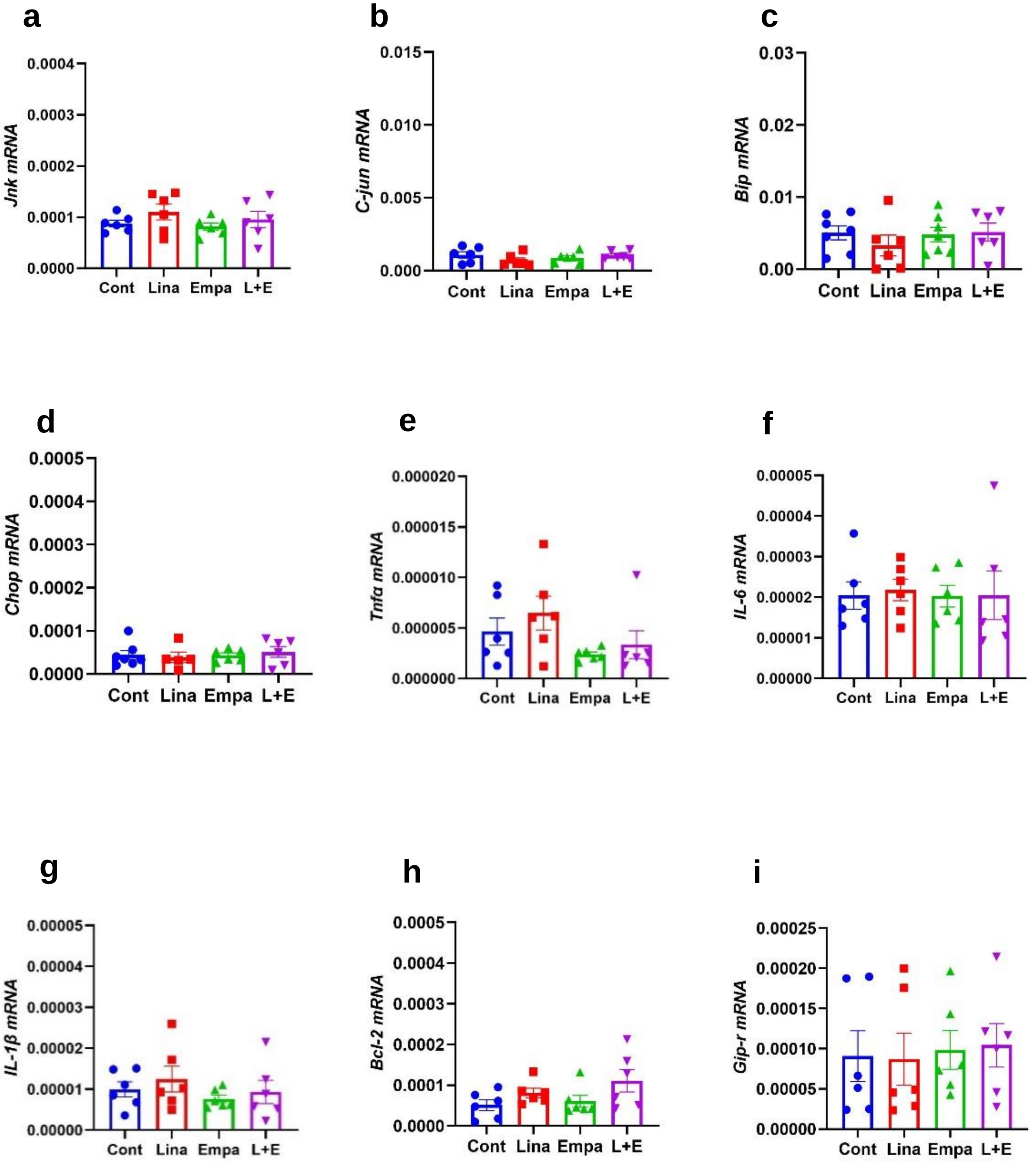

Advanced phase group

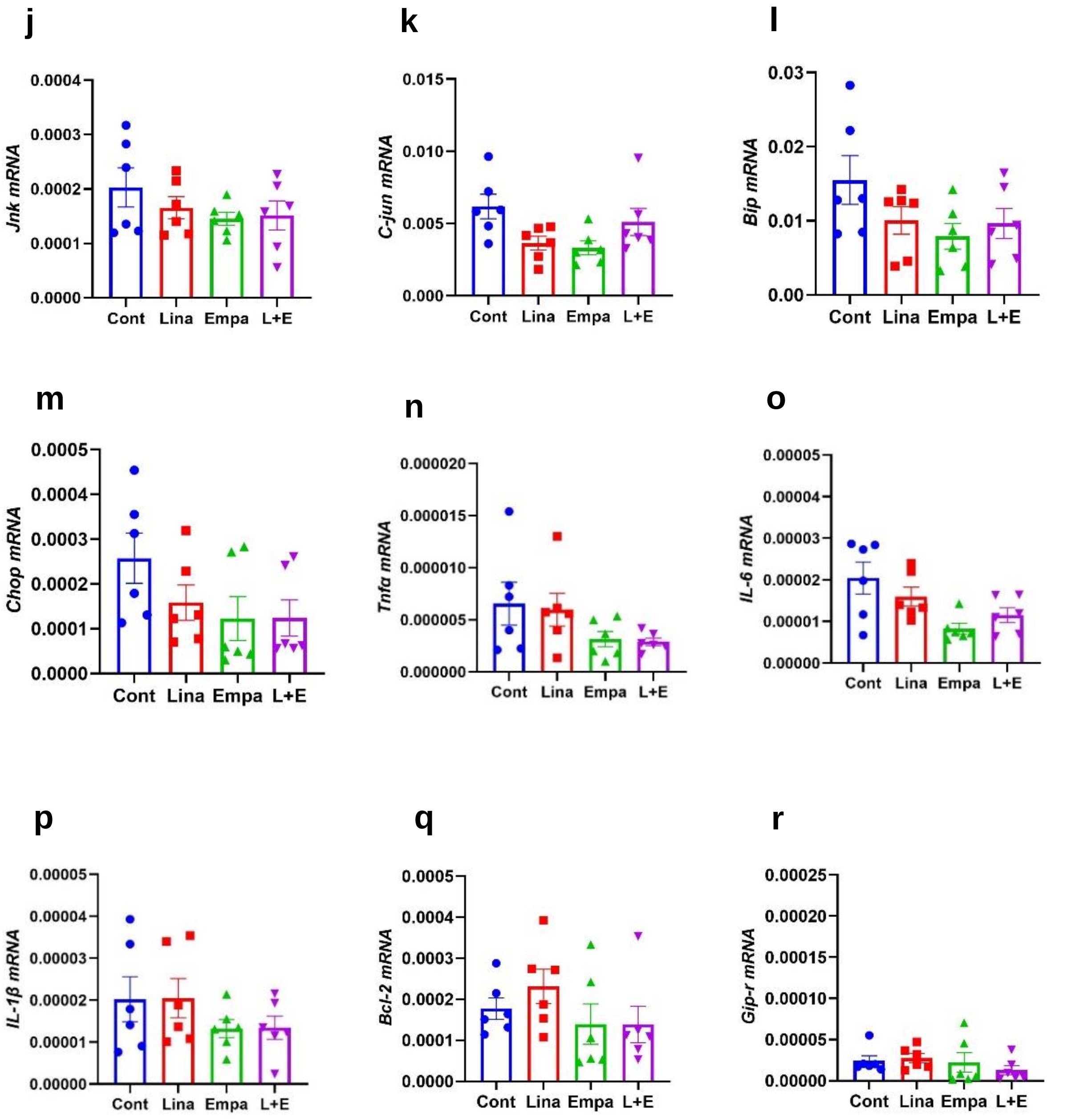

Supplementary Figure 8. Expression levels of oxidative stress, ER stress and inflammatory markers in (a–i) an early phase and (j-r) an advanced phase of diabetes. (a) (j) *Jnk*, (b) (k) *C-jun*, (c) (l) *Bip*, (d) (m) *Chop*, (e) (n) *Tnfα*, (f) (o) *Il-6*, (g) (p) *Il-1β*, (h) (q) *Bcl-2*, (i) (r) *Gip-r* (n=6). The multiple comparison was performed using the Tukey-Kramer method. Data are presented as mean ± S.D.

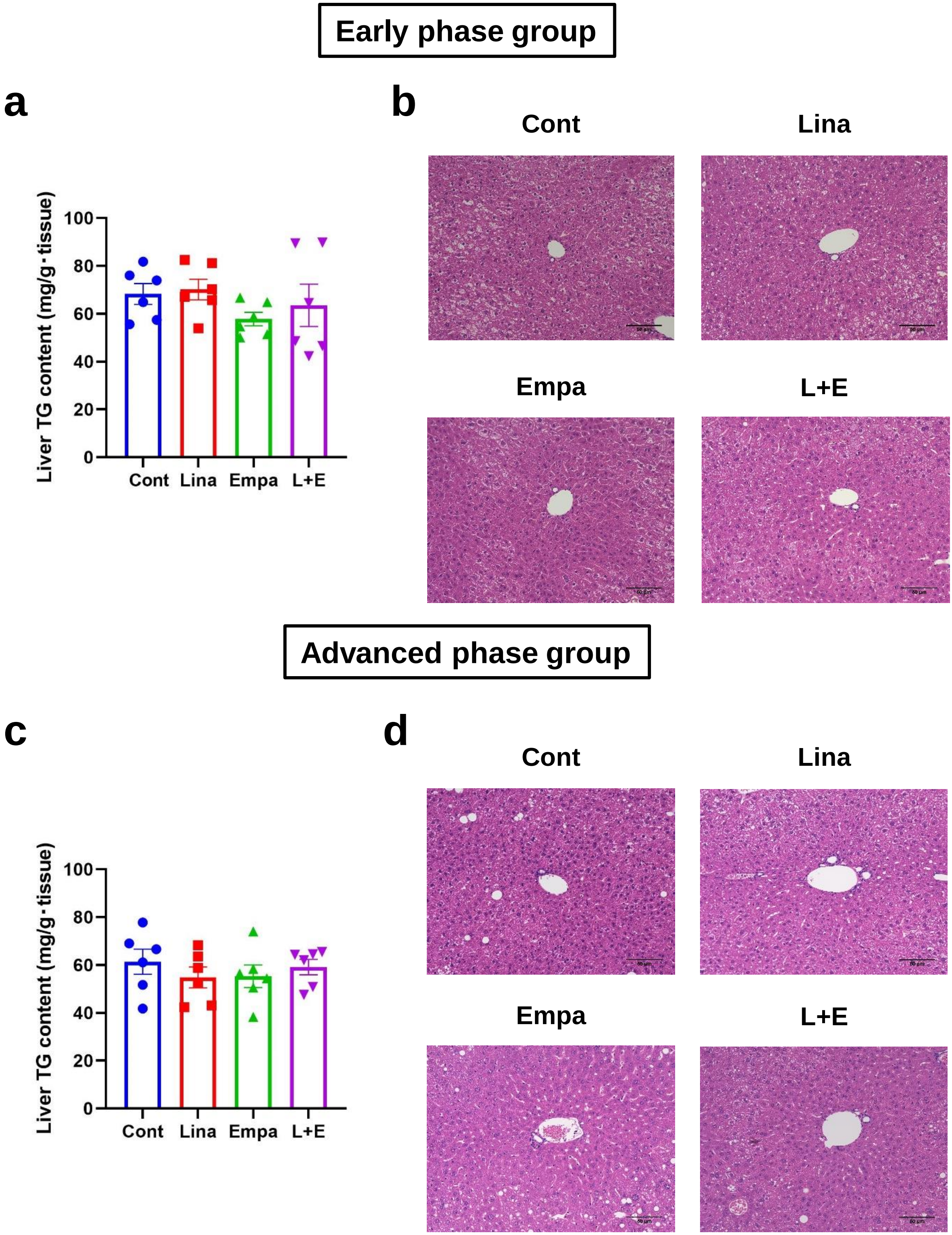

Supplementary Figure 9. HE staining of the liver and liver TG contents in (a, b) an early phase and (c, d) an advanced phase (n=6).

Early phase group

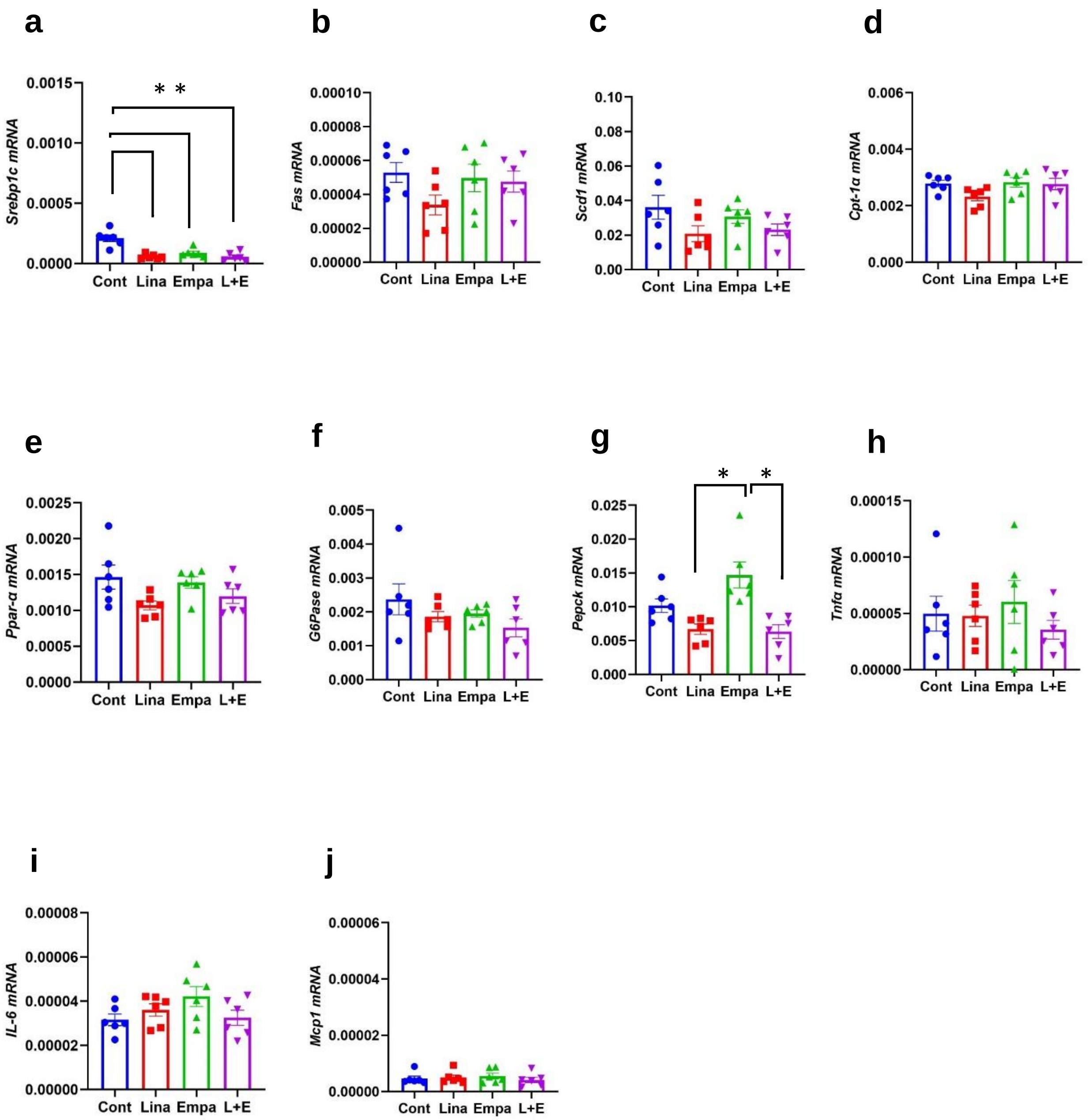

Advanced phase group

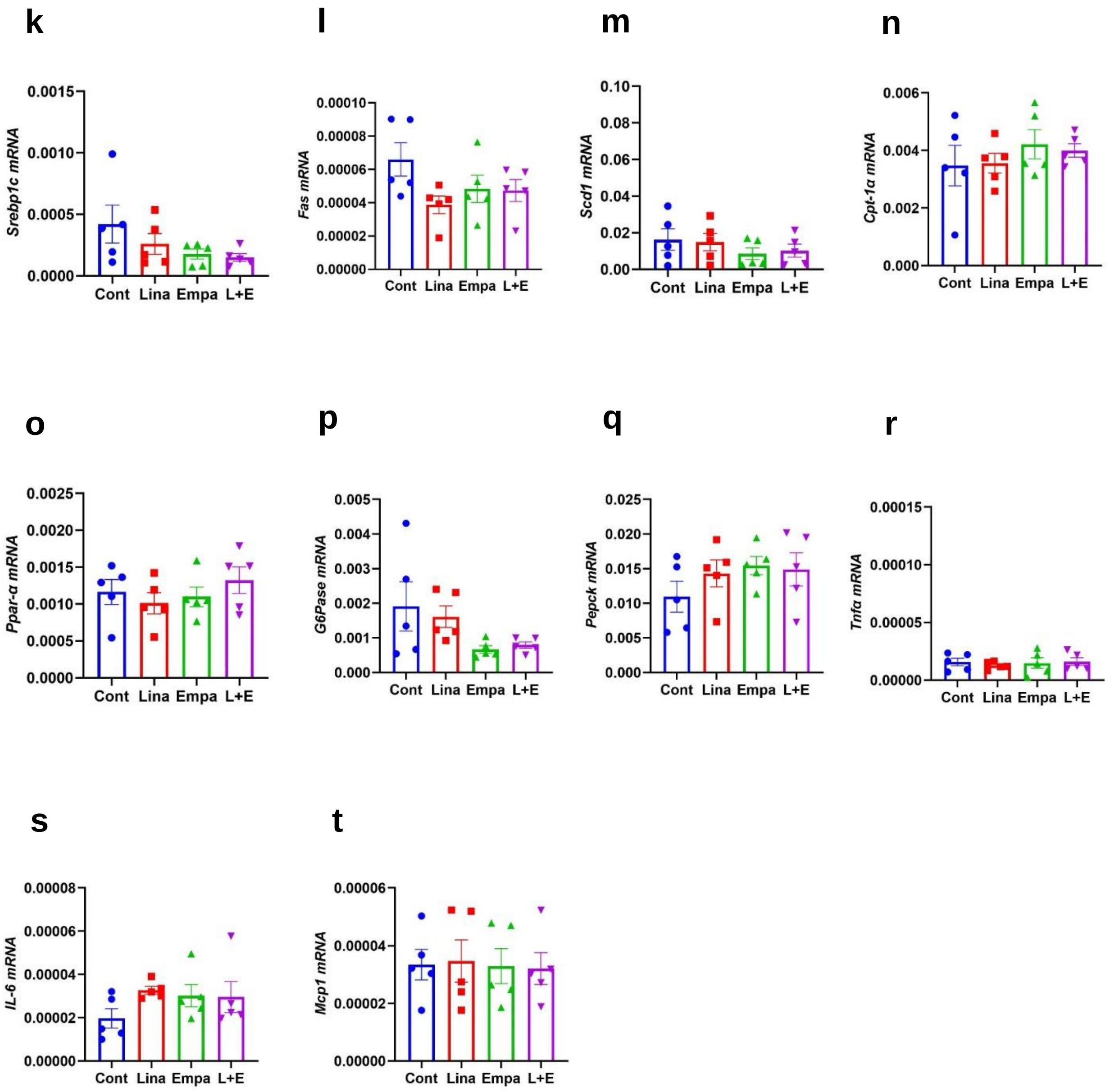

Supplementary Figure10. Expression levels of lipid synthesis, β-oxidation, gluconeogenesis and inflammatory markers in (a-j) an early phase and (k-t) an advanced phase. (a) (k) *Srebp1c*, (b) (l) *Fas*, (c) (m) *Scd1*, (d) (n) *Cpt1α*, (e) (o) *Pparα*, (f) (p) *G6Pase*, (g) (q) *Pepck*, (h) (r) *Tnfα*, (j) (s) *Il-6*, (k) (t) *Mcp1* early phase (n=6), advanced phase (n=5). The multiple comparison was performed using the Tukey-Kramer method. Data are presented as mean ± S.D.

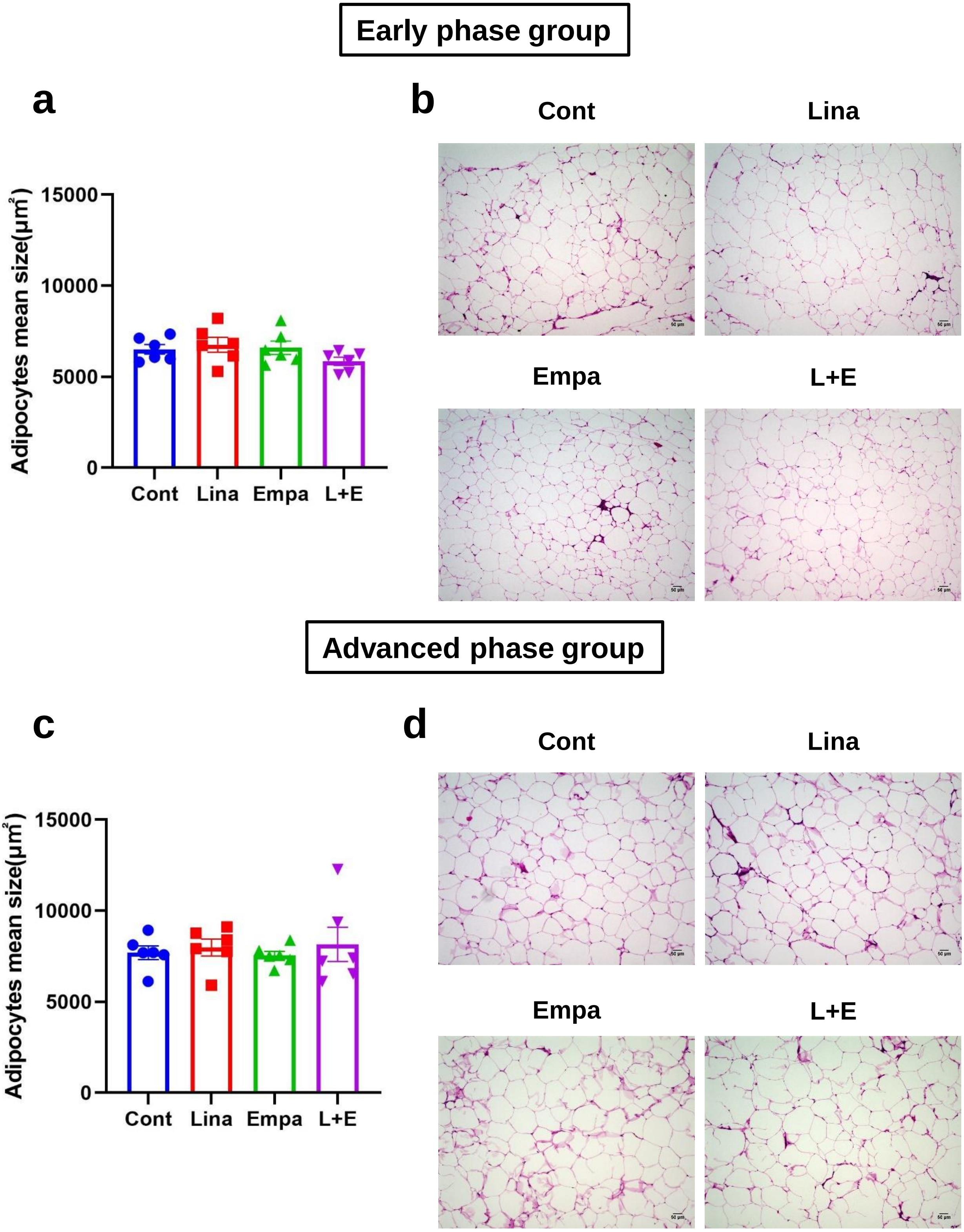

Supplementary Figure 11. HE staining of the epidydimal WAT and adipocyte size in (a, b) an early phase and (c, d) an advanced phase (n=6).

Early phase group

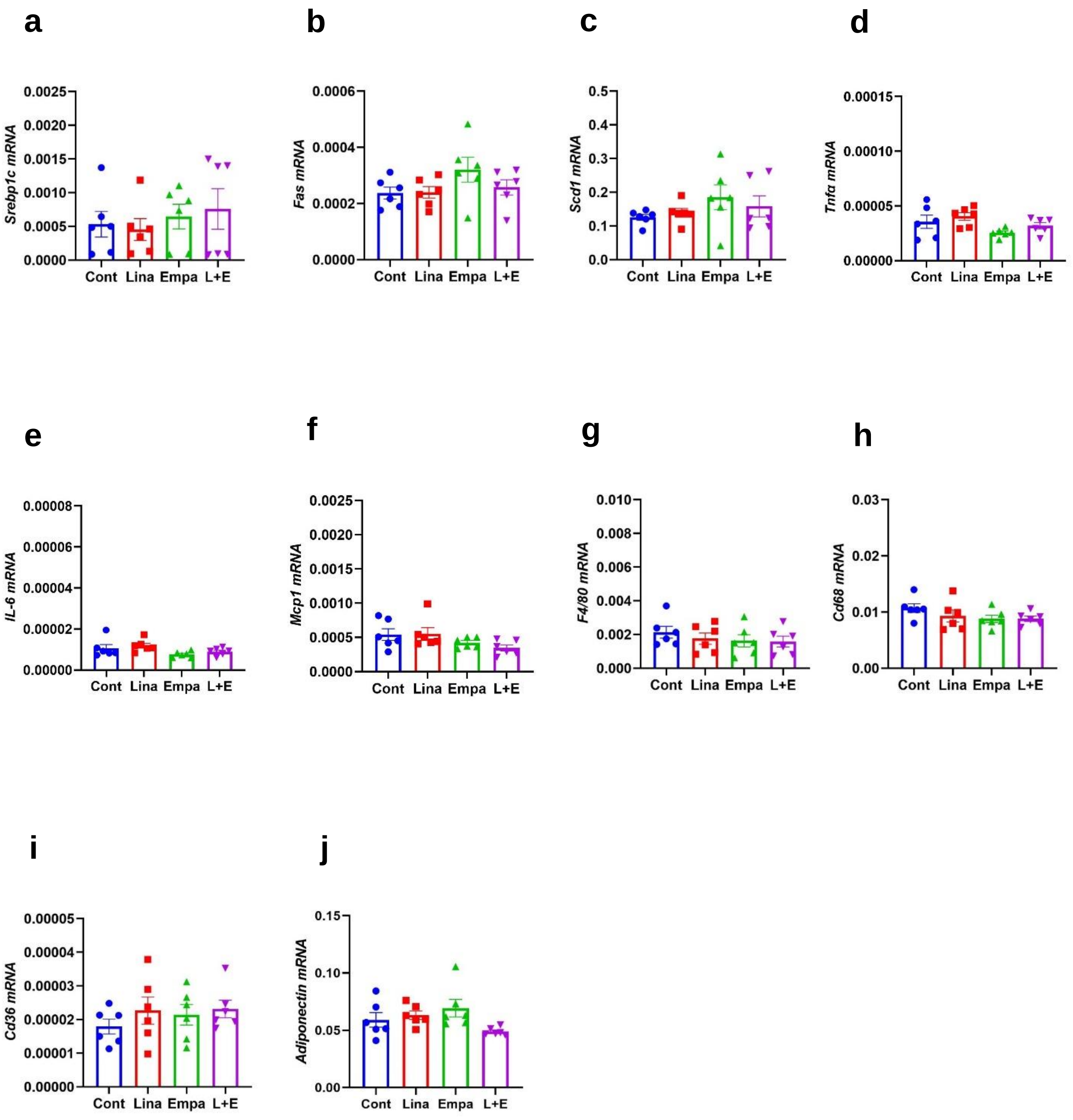

Advanced phase group

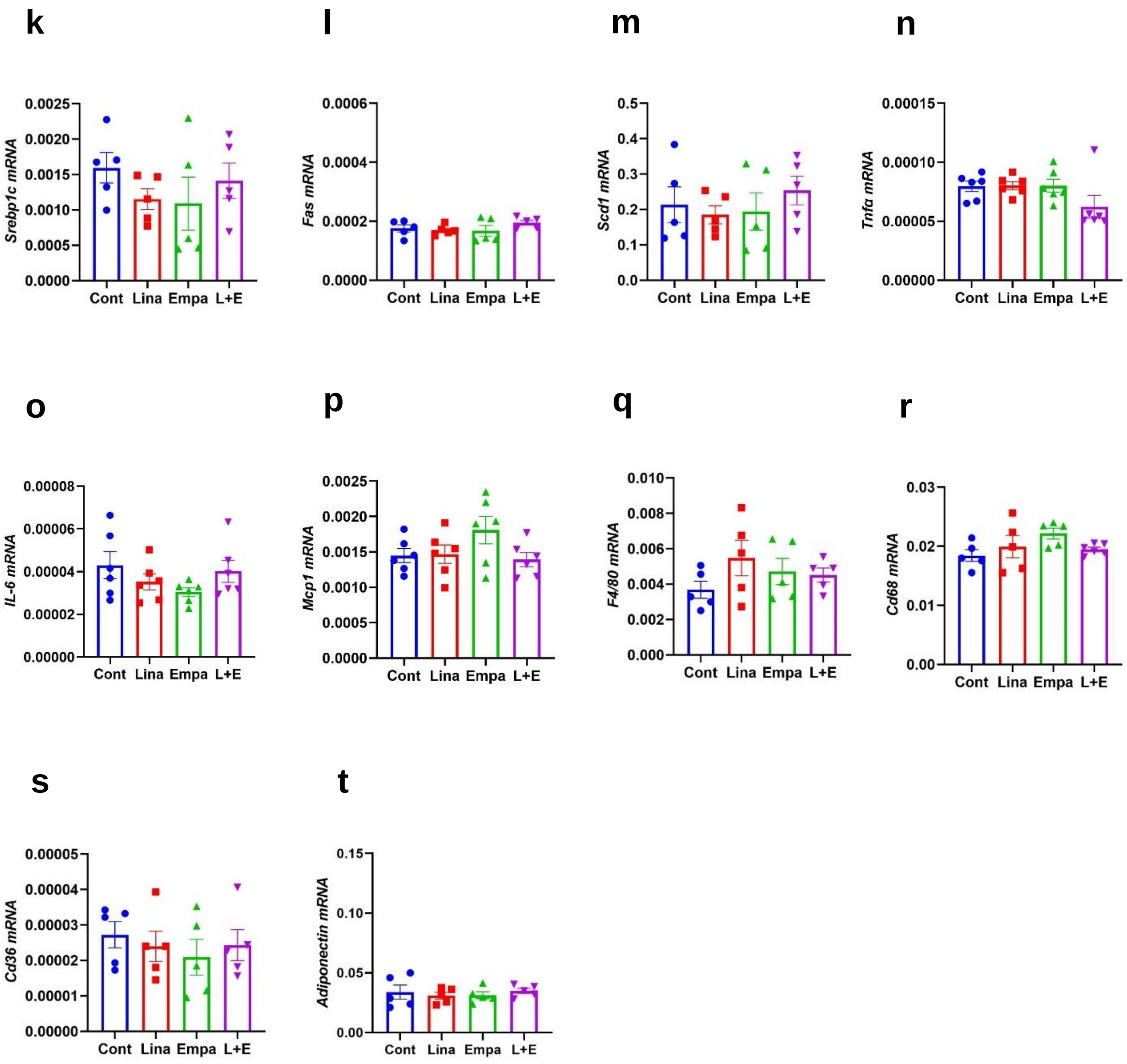

Supplementary Figure 12. Expression levels of lipid synthesis, adiponectin, macrophage markers and inflammatory markers in (a-j) an early phase and (k-t) an advanced phase. (a) (k) *Srebp1*c, (b) (l) *Fas*, (c) (m) *Scd1*, (d) (n) *Tnfα*, (e) (o) *Il-6*, (f) (p) *Mcp1*, (g) (q) *F4/80*, (h) (r) *Cd68*, (i) (s) *Cd36*, (j) (t) *Adiponectin*, early phase (n=6), advanced phase (n=5). The multiple comparison was performed using the Tukey-Kramer method. Data are presented as mean ± S.D.

Supplementary Table 1

Review of the previous reports

Literature		DPP4 inhibitor
**Hamamoto S et al. Diabetes Obes Metab. 2013** [8]	Animal	KK-A^y
	Drug	Vildagliptin (50mg/kg)
	Administration method	p.o.
	Intervention term	4 weeks from 8 weeks of age
	Glucose tolerance and insulin sensitivity	Improved glucose tolerance and insulin sensitivity
	β-cell mass	Increased
	β-cell function	Improved GSIS
	Assessment of other organs	N/A
**Mu J et al. Diabetes. 2006** [28]	Animal	ICR treated by STZ and HFD
	Drug	Des-fluoro-stagliptin
	Administration method	1.1%(premixed in HFD)
	Intervention term	12 weeks from ~10weeks of age
	Glucose tolerance and insulin sensitivity	Improved glucose tolerance, Insulin sensitivity (N/A)
	β-cell mass	Increased
	β-cell function	Improved GSIS and insulin content
	Assessment of other organs	N/A
		**SGLT2 inhibitor**
**Okauchi S et al. Biochem Biophys Res Commun. 2016** [9]	Animal	db/db
	Drug	Luseogliflozin
	Administration method	0.0025% and 0.01%(premixed in standard chow)
	Intervention term	4 weeks from 10 weeks of age
	Glucose tolerance and insulin sensitivity	Improved glucose tolerance, Insulin sensitivity (N/A)
	β-cell mass	Increased
	β-cell function	Improved GSIS and insulin content
	Assessment of other organs	N/A
**Kimura T et al. Diabetes Obes Metab. 2018** [10]	Animal	db/db
	Drug	Luseogliflozin
	Administration method	0.01%(premixed in standard chow)
	Intervention term	2weeks from 7weeks and 16weeks of age
	Glucose tolerance and insulin sensitivity	N/A
	β-cell mass	Increased
	β-cell function	Improved GSIS and insulin content
	Assessment of other organs	N/A
**Takahashi K et al. Sci Rep. 2018** [30]	Animal	db/db
	Drug	Luseogliflozin
	Administration method	0.01%(premixed in standard chow)
	Intervention term	4 weeks from 6 ,10, 14 and 24 weeks of age
	Glucose tolerance and insulin sensitivity	Improved glucose tolerance, Insulin sensitivity (N/A)
	β-cell mass	Increased
	β-cell function	Improved GSIS and insulin content
	Assessment of other organs	N/A
**Macdonald FR et al. Diabetes Obes Metab. 2010** [36]	Animal	Female Zucker diabetic fatty rats
	Drug	Dapagliflozin
	Administration method	1mg/kg p.o. in water fed HFD
	Intervention term	5 weeks from 7~8-week of age
	Glucose tolerance and insulin sensitivity	Glucose tolerance (N/A), Improved insulin sensitivity index
	β-cell mass	No change in mass with preserved islet morphology
	β-cell function	Improved disposition index
	Assessment of other organs	N/A

Supplementary Table 2

Primer Sequences

Genes	Forward	Reverse
18sr	GCGCTTCCTTACCTGGTTGAT	GCCATTCGCAGTTTCACTGTAC
Ins1	CCCTTAGTGACCAGCTATAATCAGAGA	ACCACAAAGATGCTGTTTGACAA
Ins2	CTGCTGGCCCTGCTCTTC	AACCACAAAGGTGCTGCTTGA
Mafa	CCAGCTGGTATCCATGTCC	TTCTGTTTCAGTCGGATGACC
Pdx-1	TTCCCGAATGGAACCGAGCCTG	TTTTCCTCGGGTTCCGCTGTGT
NeuroD	AGGAACACGAGGCAGACAAGA	CTCCCCCGTTTCTCAGAGAGT
Nkx6.1	CAAACCTCTGGACCCGAACTC	GCTGCCACCGCTCGATT
Irs2	CATCGACTTCCTGTCCCATCA	CCCATCCTCAAGGTCAAAGG
Glp-1r	ACTTTCTTTCTCCGCCTTGGT	CCTGGTGCAGTGCAAGTGTCT
Gip-r	GGAGCGCAACGAAGTCAAA	CTGGCCCTACCAAGATGGTTAT
Jnk	AGGGCAGCCGTCTCCTTTAG	GGCGAAGACGATGGATGCT
C-jun	GAAACGACCTTCTACGACGAT	GAATCTTAGGGTTACTGTAGCCGTA
Bip	TCATCGGACGCACTTGGAA	AACCACCTTGAATGGCAAGAA
Chop	GGAGCCAGGGCCAACAG	GCCATAGAACTCTGACTGGAATCTG
Tnf-α	TGATCCGCGACGTGGAA	ACCGCCTGGAGTTCTGGAA
IL-6	CTTCCTACCCCAATTTCCAATG	GAATTGGATGGTCTTGGTCCTTA
IL-1β	TCGCTCAGGGTCACAAGAAA	CATCAGAGGCAAGGAGGAAAAC
Collagen1	CAACCTGGACGCCATCAAG	CAGACGGCTGAGTAGGGAACA
Collagen4	GGTTTGGCAGGCTCTTGTCTT	CTGGGCGTAGTGGCACACTT
Fibronectin	ACCAACCTTAATCCGGGCAC	TCAGAAACTGTGGCTTGCTGG
Fas	TGCATGACAGCATCCAAGACA	CTCTTCCCATGAGATTGGTACCA
Scd1	CAACACCATGGCGTTCCA	GGTGGGCGCGGTGAT
Srebp1c	GGGAGGACCCAAGGTGACA	ACACGGACGGGTACATCTTTAAA
Ppar-α	CGATGCTGTCCTCCTTGATGA	TTCTTAAGGAACTCGCGTGTGA
Cpt-1α	CCTGGGCATGATTGCAAAG	ACGCCACTCACGATGTTCTCC
G6Pase	AAGAGACTGTGGGCATCAATCTC	ATCCACTTGAAGACGAGGTTGAA
Pepck	CCACAGCTGCTGCAGAACAC	GAAGGGTCGCATGGCAAA
Mcp1	CTTCCTCCACCACCATGCA	CCAGCCGGCAACTGTGA
Adiponectin	CAGTGGATCTGACGACACCAA	TGGGCAGGATTAAGAGGAACA
Cd36	GGAGCCACTGGTCGTTGAA	TGATTAGCCCGGTGCCTTTA
Cd68	GGACTACATGGCGGTGGAATA	GATGAATTCTGCGCCATGAA
F4/80	TGCATCTAGCAATGGACAGC	GCCTTCTGGATCCATTTGAA
